# Nanoparticle and Bioparticle Deposition Kinetics: Quartz Microbalance Measurements

**DOI:** 10.3390/nano11010145

**Published:** 2021-01-08

**Authors:** Anna Bratek-Skicki, Marta Sadowska, Julia Maciejewska-Prończuk, Zbigniew Adamczyk

**Affiliations:** 1 Structural Biology Brussels, Vrije Universiteit Brussel, Pleinlaan 2, 1050 Brussels, Belgium; 2 Jerzy Haber Institute of Catalysis and Surface Chemistry, Polish Academy of Sciences, Niezapominajek 8, 30-239 Krakow, Poland; marta.sadowska@ikifp.edu.pl (M.S.); zbigniew.adamczyk@ikifp.edu.pl (Z.A.); 3 Department of Chemical and Process Engineering, Cracow University of Technology, Warszawska 24, PL-31155 Krakow, Poland; julia.maciejewska-pronczuk@pk.edu.pl

**Keywords:** deposition of bioparticles, modeling of particle deposition, nanoparticle deposition, protein adsorption, quartz microbalance measurements, virus attachment

## Abstract

Controlled deposition of nanoparticles and bioparticles is necessary for their separation and purification by chromatography, filtration, food emulsion and foam stabilization, etc. Compared to numerous experimental techniques used to quantify bioparticle deposition kinetics, the quartz crystal microbalance (QCM) method is advantageous because it enables real time measurements under different transport conditions with high precision. Because of its versatility and the deceptive simplicity of measurements, this technique is used in a plethora of investigations involving nanoparticles, macroions, proteins, viruses, bacteria and cells. However, in contrast to the robustness of the measurements, theoretical interpretations of QCM measurements for a particle-like load is complicated because the primary signals (the oscillation frequency and the band width shifts) depend on the force exerted on the sensor rather than on the particle mass. Therefore, it is postulated that a proper interpretation of the QCM data requires a reliable theoretical framework furnishing reference results for well-defined systems. Providing such results is a primary motivation of this work where the kinetics of particle deposition under diffusion and flow conditions is discussed. Expressions for calculating the deposition rates and the maximum coverage are presented. Theoretical results describing the QCM response to a heterogeneous load are discussed, which enables a quantitative interpretation of experimental data obtained for nanoparticles and bioparticles comprising viruses and protein molecules.

## 1. Introduction

Effective deposition of particles on various surfaces is important for many practical processes, such as water and wastewater filtration, flotation, separation of toner and ink particles, coating formation, paper making, catalysis, colloid lithography, food emulsion and foam stabilization, etc. Particularly significant is the deposition of noble metal particles, which find applications in biology, medicine, chemical analysis, electronic, cosmetic and pharmaceutical industries [1,2,3,4,5,6]. For example, silver nanoparticles, owing to their pronounced biocidal properties, are applied to modify surfaces of various consumer products such as clothes, laboratory and surgical gowns, dressing bandages, etc. [7,8]. They also serve as analytical sensors in surface enhanced Raman spectroscopy (SERS) and metal-enhanced fluorescence (MEF) [9,10]. A large surface area and surface energy make the noble metal and other nanoparticles such as silica, titanium, zinc and copper oxides valuable materials for catalysis [11,12].

In addition to the wide range of applications, investigations of particle deposition furnish essential information about their interactions with interfaces, e.g., those mimicking cell membranes [13], which is a crucial issue for colloid science, biophysics, medicine, soil chemistry, etc.

Analogously, a controlled deposition of bioparticles such as protein molecules, viruses, bacteria and cells is necessary for their efficient separation and purification by chromatography and filtration for biosensing, bioreactors, immunological assays, etc. Determining virus attachment to various substrates (e.g., metals) is essential for devising strategies for their efficient deactivation and removal.

On the other hand, undesired protein adsorption can result in artificial organ and implant failure, plaque formation, inflammatory response, blocking of sensors, and the fouling of ultrafiltration units.

Because of its significance, particle and bioparticle deposition has been extensively studied, using a plethora of experimental methods, such as the optical microscopy [14,15,16,17], scanning probe microcopies comprising atomic force microscopy (AFM) [18,19,20,21], scanning electron microscopy (SEM) [22,23], optical techniques such as ellipsometry, reflectometry [24,25], light mode waveguide spectroscopy (OWLS) [26,27,28,29] and surface plasmon resonance (SPR) [30,31]. However, these techniques exhibit some limitations; in particular, they do not provide information about the adhesive contact strength among the deposited particles and the substrate.

There exist other precise methods for determining particle and protein adsorption that are based on electrokinetic measurements, most frequently of the streaming current or the streaming potential [14,15,18,32,33,34,35]. These methods enable direct in situ measurements of the deposition and desorption kinetics, yielding interesting information about the influence of various physicochemical parameters on the binding energy of solutes. However, the disadvantage of the electrokinetic methods is their limited sensitivity for larger particle coverage and for slightly charged solutes substrates.

Compared to the above-mentioned techniques, the quartz crystal microbalance (QCM) method is advantageous enabling real time measurements of particle adsorption/desorption kinetics under different transport conditions with a high precision. Moreover, in contrast to the streaming potential methods, the QCM signal is not directly affected by the solute or the interface charge. Because of its numerous advantages and a deceptive simplicity of measurements, the QCM method is used in numerous investigations involving nanoparticles [3,36,37,38,39,40,41,42,43,44,45,46,47,48,49,50], macroions [29,31,51,52,53] proteins [27,28,54,55,56,57,58,59,60,61,62,63,64,65], viruses [66,67,68,69,70], bacteria [71,72,73,74,75,76,77], and living cells [78,79,80,81,82].

One should emphasize, however, that in contrast to the robustness of performing experiments, a theoretical interpretation of QCM measurements for a particle-like load is a challenging task [83,84]. The main source of misinterpretations of experimental data is that the primary signals, i.e., the oscillation frequency and the band width (dissipation) shifts, depend on the force exerted on the sensor rather than on the particle mass. In the case of a rigid contact, where the particles are firmly fixed at the sensor, the net force consists of the inertia component proportional to the particle mass and the hydrodynamic component. The latter depends on many parameters, such as the solvent density and viscosity, the particle size and shape, its orientation on the surface, the particle coverage, the sensor oscillation frequency (overtone number) and the sensor topography, primarily its roughness [84].

The situation becomes even more involved if the particle/substrate adhesion strength is not sufficient to ensure a rigid contact, allowing for particle oscillatory or rolling motions on the surfaces [38,48,50,83]. The additional hydrodynamic and elastic forces, which appear because of particle motions, modify the QCM signals, especially for particle sizes above 100 nm. As a result, the measured frequency shift becomes much smaller (in absolute terms) than for the rigid contact regime and it may even become positive which yields an apparent negative mass of microparticles [38].

Considering these facts, one can postulate that a proper interpretation of QCM data requires a careful analysis of the influence of all the above-mentioned parameters and a reliable theoretical framework furnishing reference results for some well-controlled systems such as spherical particles under the rigid contact regime. Providing such results is a primary motivation of this work, which is organized as follows: in Section 2, the kinetics of particle deposition on planar interfaces under diffusion and flow conditions are discussed, and the limiting solutions for the initial deposition rates and the maximum coverage are presented. In Section 3, theoretical results describing the QCM response to a heterogeneous (particle-like) load are discussed. This enables a quantitative interpretation of experimental data obtained for nanoparticles and bioparticles, comprising viruses and protein molecules, which are presented in the Section 4 of our work. In Section 5, conclusions summarizing the presented results are presented.

## 2. Kinetics of Particle Deposition—Theoretical Aspects

### 2.1. General Considerations

Deposition of particles on solid substrates under flow conditions is a complex process consisting of three major steps: transfer of particles from the bulk of the suspension to the immediate vicinity of boundary surfaces, transfer of particles through the surface boundary layer adjacent to the interface, and the formation of a permanent adhesive contact with the interface or previously deposited particles leading to particle immobilization.

Particle transfer in the first step is governed by forced convection (mixing) or natural convection and by external forces, primarily the gravitational force inducing the sedimentation effect. At distances comparable with particle dimensions the diffusion becomes the most significant transport mechanism. At still smaller distances in the range of 10 nm, the particle motion is controlled by specific forces mainly consisting of the electric double-layer and the van der Waals interactions whose magnitude is affected by the particle shape, elasticity modulus, and the substrate’s (sensor’s) roughness.

There are two major parameters which quantitatively characterize particle deposition kinetics on solid substrates: the mass transfer rate constant *k_c_*, characterizing the initial deposition rate where the blocking effects are negligible, and the maximum coverage (referred as the jamming coverage) mainly governed by the particle shape, orientation in the layer, and the substrate topography.

The initial deposition kinetics can be efficiently analyzed in terms of the convective diffusion theory formulated by Levich [85] and extended in other works [86,87,88,89]. On the other hand, the more complicated problem of predicting the maximum, mono- and multilayer coverage can be effectively treated in terms of various coarse-graining Monte-Carlo approaches [90,91,92,93,94].

### 2.2. Limiting Solutions—Initial Deposition Rates

The particle or bioparticle transfer from the bulk to a solid substrate can be described by the convective-diffusion theory exploiting the continuity (mass conservation) equation, referred to as the Smoluchowski–Levich equation [89]

(1) $$\frac{\partial \;n}{\partial \;t}=D\;\nabla^{2}n-\frac{D}{kT}\nabla \cdot \left(F\;n\right)\;-V\cdot \nabla n$$

where *n* is the particle number concentration in the suspension, *t* is the time, *D* is the particle diffusion coefficient, *k* is the Boltzmann constant, *T* is the absolute temperature, **F** is the external force vector, and **V** is the fluid velocity vector.

One should mention that by deriving Equation (1), all hydrodynamic and specific interactions among particles are neglected. Moreover, the diffusion coefficient is assumed to be independent of the particle concentration and its gradient. As a consequence, Equation (1) is strictly valid for diluted suspensions of non-interacting particles.

If the external force **F** and the flow vanish, the system is purely under diffusion-controlled transport, and the Smoluchowski equation simplifies to the form

(2) $$\frac{\partial \;n}{\partial \;t}=D\;\nabla^{2}n$$

This equation, which is linear in respect to the particle concentration, can be analytically solved, using, for example, the Laplace transformation method, for many situations of practical interest, e.g., for planar interfaces in the contact with stagnant suspensions of a finite extension [89].

On the other hand, assuming steady-state conditions and neglecting external forces, Equation (1) simplifies to the form

(3) $${\bar{\nabla }}^{2}\;n-Pe\;\bar{V}\cdot \bar{\nabla }\;n=0$$

where $Pe=\frac{\;V_{ch}\;L_{ch}\;}{D_{}}$ is the dimensionless Peclet number characterizing the ratio of the flow to the diffusion rates, $\bar{\nabla }=L_{ch}\;\nabla ,\;\bar{V}=\frac{1}{V_{ch}}\;V$, where $L_{ch}$ is the characteristic length scale and *V_ch_* is the characteristic convection velocity.

In a general case, particle deposition kinetics can be evaluated integrating Equations (1)–(3) with appropriate boundary conditions and rigorously taking into account the hydrodynamic and specific interactions. Solutions of these equations are available for several interface (sensors) geometries of practical significance such as planar, cylindrical, and spherical using simplified form of the boundary conditions at the interface [89]. One of the important forms of the boundary condition appears if all particles arriving at the sensor become irreversibly attached, although they may undergo lateral motion over the interface. This case corresponds to the perfect sink model originally introduced by Smoluchowski to describe particle aggregation phenomena [95], formulated as

(4) $$n=0\;\mathrm{at}\;z=\delta_{m}$$

where $\delta_{m}$ is the primary minimum distance, and *z* is the separation distance from the interface.

Using this condition, interesting analytical solutions describing particle deposition rates can be derived for various situations of practical significance. For example, in the case of diffusion-controlled deposition of particles at a planar interface from an infinite volume, their flux is described by the formula derived from Smoluchowski [95]

(5) $$-j_{b}=\;{\left(\frac{\;D_{}\;}{\pi \;t}\right)}^{1/2}n_{b}$$

where $-j_{b}$ is the particle flux directed to the substrate, i.e., opposite to the direction of the *z* axis, and $n_{b}$ is the uniform concentration of the particle in the bulk.

Equation (5) indicates that the flux vanishes proportionally to *t*^−1/2^, which means that particle adsorption at planar interfaces, driven by diffusion alone, becomes ineffective for long time periods.

Integrating Equation (5), one obtains the expression for surface concentration of adsorbed particles

(6) $$N=2\;{\left(\frac{D\;t}{\pi }\right)}^{1/2}n_{b}$$

For the interpretation of QCM measurements, it is convenient to express Equation (6) in terms of the mass per unit area (coverage), which is connected with the surface concentration by the linear relationship

(7) $$\Gamma =N_{}m_{p}$$

where $m_{p}$ is the mass of a single particle.

In this way, Equation (6) becomes

(8) $$\Gamma =2\;{\left(\frac{D\;t}{\pi }\right)}^{1/2}c_{b}$$

where $c_{b}=n_{b}m_{p}$ is the mass concentration of particles often expressed in mg L^−1^ (ppm).

The analogous solution for a cell composed of two flat plates of a size considerably larger than their separation distance *h* has the following form [89]

(9) $$-j_{b}=\frac{2D}{h}n_{b}\sum_{l=1}^{\infty }e^{-\;\frac{{\left(2l\;-1\right)}^{2}\pi^{2}Dt}{4h^{2}}}\;\Gamma =h\left[1-\frac{8}{\pi^{2}}\sum_{l=1}^{\infty }\frac{e^{-\;\frac{{\left(2l\;-1\right)}^{2}\pi^{2}Dt}{4h^{2}}}}{{\left(2l\;-1\right)}^{2}}\right]c_{b}$$

These solutions, yielding the maximum flux and deposition rates available under diffusion-controlled transport, are useful reference data for a quantitative analysis of experimental particle deposition kinetics. If smaller flux is experimentally determined, one can expect particle aggregation in the bulk or that the sensor surface is not fully available for particles due to contamination. Another explanation of smaller flux than these limiting values is an insufficiently strong adhesion of particles to sensors allowing for their desorption.

Because the diffusion transport in the case of planar interfaces becomes ineffective for longer time, in order to increase the deposition rate, the experiments are usually carried out under a forced convection regime creating flows of desired configuration and intensity, as is the case for QCM cells. Additionally, under convection-driven transport, the transient time of establishing steady-state conditions is usually much shorter than the time of experiments. Therefore, one can use to describe particle deposition rates the stationary convective diffusion equation, Equation (3). It can be simplified for many situations of practical interest to one-dimensional forms, if the perpendicular fluid component is independent of the position over the sensor. In this case, the particle deposition kinetics becomes a linear function of time

(10) $$\Gamma =k_{c}c_{b}t$$

where the deposition rate constant *k_c_* can be calculated from the formula [89,96]

(11) $$k_{c}=C_{f}\frac{D}{L_{ch}\;}\;Pe^{1/3}$$

where $C_{f}$ is the dimensionless constant depending on the flow configuration.

Analytical expressions for *k_c_* are given in Ref. [89] for various flows.

Considering that $Pe=\frac{\;V_{ch}\;L_{ch}\;}{D_{}}$ one can invoke from Equation (11) that *k_c_* increases proportionally to $D_{}^{2/3}$, which means that the convective flux decreases as $a^{-2/3}$ with particle size. It is also interesting to observe that *k_c_* is rather insensitive to the characteristic fluid velocity $V_{ch}$, increasing in all cases proportionally to $V_{ch}^{1/3}$.

For experimental cells in the form of parallel-plate channels, the mass transfer rate depends on the distance from the inlet point and is given by the formula [96]

(12) $$k_{c}=0.776\;\frac{V_{ch}^{1/3}\;D^{2/3}}{b^{2/3}\;{\bar{x}}^{1/3}}$$

where *b* is the half depths of the channel, $\bar{x}=x/b$ is the dimensionless distance from the inlet to the channel, *L* is the channel length and

(13) $$V_{ch}=\frac{Q}{2bl}=\frac{P\;b^{2}}{3\eta \;L}$$

is the averaged fluid velocity in the channel, *P* is the hydrostatic pressure drop along the channel, *η* is the dynamic viscosity of the fluid, *Q* is the volume flow rate of the suspension and *l* is the channel width.

The mass transfer rate constant averaged over the entire channel surface area denoted as $\langle k_{c}\rangle$ is given by [89]

(14) $$\langle k_{c}\rangle =0.924\;\frac{Q_{}^{\;1/3}\;D{\;}^{2/3}}{b^{2/3}\;l^{\;1/3}L^{\;1/3}}$$

As shown in Refs. [3,97], an analogous expression is also applicable for the particle deposition on QCM sensors where the flow pattern is somewhat intermediate between the impinging-jet and the channel flows, thus

(15) $$\langle k_{c}\rangle =k_{c0}\;Q_{}^{\;1/3}\;D{\;}^{2/3}$$

where the $k_{c0}$ is the mass transfer rate constant for a particular QCM cell, which can be derived from numerical modeling or from calibrating experiments involving gold nanoparticles of large density [3].

It is important to mention, however, that the above analytical expressions for the mass transfer rates are strictly valid for nanoparticle size ranges. For larger particles the kinetics of their transfer to interfaces can be more accurately predicted solving the governing mass-balance equation, Equation (3), which considers the effects of interception, and specific, external and hydrodynamic forces in an exact manner. Numerical solutions of this equation for collectors of practical interest are discussed elsewhere [89].

One should emphasize that the above solutions for the convective transport conditions yield the maximum flux and deposition rates, analogously, as for the diffusion-controlled transport.

### 2.3. Surface Blocking Effects—Maximum Coverages

The above solutions characterize the deposition kinetics neglecting the desorption and the blocking effects appearing because of the steric interactions of the adsorbing particle with those attached to the interface. To describe particle deposition kinetics in this case, the surface boundary layer (SFBL) method was developed in Refs. [42,98] assuming continuity of the particle flux. Thus, by virtue of this assumption, the particle flux from the bulk *j_b_* discussed above is equal to the particle flux through the adsorption boundary layer *j_a_* of the thickness *δ_a_*. The adsorption flux is calculated assuming a negligible convection in the adsorption layer and considering the blocking effects. It can be expressed in the following form [89]

(16) $$\;j_{a}\;=\;\frac{1}{S_{g}}\frac{d\Theta }{dt}=\;k_{a\;}n(\delta_{a})\;B(\Theta )-\frac{k_{d}}{S_{g}}\Theta$$

where *j_a_* is the net adsorption/desorption flux, $S_{g}$ is the characteristic cross-section of the particle, $\Theta =S_{g}N$ is the absolute particle coverage, $\;k_{a\;},\;k_{d}$ are the adsorption and desorption constants, $n(\delta_{a})$ is the number concentration of particles at the adsorption boundary layer and B(Θ) is the generalized blocking function, more appropriately referred to as the available surface function.

Equation (16) serves as the general kinetic boundary condition for the bulk transport equation, Equations (2) and (3). However, since it is nonlinear with respect to the particle coverage its analytical solution in the case of diffusion-controlled transport is not feasible. The situation simplifies under convective one-dimensional transport, where the particle concentration $n(\delta_{a})$ remains in a local equilibrium with the surface coverage. In this case, the constitutive expression for the adsorption flux, Equation (16) becomes [89]

(17) $$j_{a}=\frac{\;K\;B(\Theta )-K_{d}\Theta }{(K-1)B(\Theta )+1}k_{c}n_{b}$$

where *K* = *k_a_/k_c_* is the dimensionless coupling constants, $K_{d}=k_{d}/(S_{g}k_{c}n_{b})$ is the dimensionless desorption constant, *k_c_* is the bulk transfer rate constant discussed above, and *n_b_* is the bulk number concentration of particles.

Equation (17) can be integrated, which yields the following dependence

(18) $$\int_{\Theta_{0}}^{\Theta }\frac{\;(K-1)\;B\left(\Theta^{\prime }\right)+1}{K\;B\left(\Theta^{\prime }\right)-K_{d}\Theta^{\prime }}\;d\Theta^{\prime }=S_{g}k_{c}n_{b}t$$

where $\Theta_{0}$ is the initial coverage of particles.

Equation (18) represents a general solution for particle deposition kinetics under convection driven transport. However, it can only be evaluated by numerical integration if the blocking function is known in an analytical form.

As before, to analyze the QCM experimental data, it is useful to express Equation (18) in terms of the mass coverage of particles connected with the absolute coverage by

(19) $$\Gamma =(\Theta /S_{g})m_{1}$$

Using this definition, one obtains

(20) $$\int_{\Gamma_{0}}^{\Gamma_{}}\frac{\left(k_{a}-k_{c}\right)B\left(\Gamma^{\prime }\right)+k_{c}}{k_{a}c_{b}B\left(\Gamma^{\prime }\right)-k_{d}\Gamma^{\prime }}d\Gamma^{\prime }=k_{c}t$$

In a general case, Equation (20) can be solved by numerical integration if the kinetic constants, the blocking function and the maximum coverage are known. However, for a bulk transport regime characterized by the condition $k_{a}>>k_{c}$ and a lower coverage range, it simplifies to the previously derived linear form, Equation (10).

The adsorption and the desorption constants in Equation (20) are given in the general case by the expressions [89]

(21) $$k_{a}^{}=\frac{e^{\;\phi \left(\delta_{a}\right)/kT}}{\int_{\delta_{m}}^{\;\delta_{a}}\frac{\;e^{\;\phi \left(z^{\prime }\right)/kT}}{D\left(z^{\prime }\right)}\;dz^{\prime }}\;\;k_{d}^{\;}=k_{a}^{\;}\;e^{\;\left[\phi \left(\delta_{m}\right)-\phi \left(\delta_{a}\right)\right]/kT}$$

where $\phi \left(\delta_{a}\right)$ is the interaction energy at the distance *δ_a_*, *D*(*z*′) is the diffusion coefficient of the particles in the adsorption layer, which depends on the distance from the substrate surface, and $\phi \left(\delta_{m}\right)$ is the specific interaction energy of the particle with the interface at *z* = *δ_m_* (the primary minimum distance).

Knowing the interaction energy around the primary minimum and the barrier height one can evaluate the kinetic adsorption and desorption constants according to the method applied in Ref. [98].

For a barrierless adsorption regime, the kinetic adsorption constant can be calculated from the dependence [89]

(22) $$k_{a}={\frac{D}{\delta_{a}^{}[1+0.5_{}^{}\ln(\delta_{a}^{}/\delta_{m}^{})]}}_{}$$

Additionally, to explicitly calculate particle deposition kinetics from Equations (18) and (20), the blocking function is needed, which can be conveniently acquired from the random sequential adsorption (RSA) modeling [90,91,93,94,99,100]. For moderate particle coverage, one can approximate the blocking function by the second order polynomial expansion [93]

(23) $$B\left(\Theta_{}\right)=1-\;C_{1}\;\Theta_{}+C_{2}\;\Theta_{}^{2}+0\left(\Theta_{}^{\;3}\right)$$

For spherical particles $C_{1}=4$ and $C_{2}=6\sqrt{3}/\pi =3.31$.

On the other hand, for coverages approaching the jamming coverage $\Theta_{\infty }$, which is equal to 0.547 for spherical particles [100], the blocking function can be approximated by the expression

(24) $$B\left(\Theta_{}\right)=2.31\;{\left(1-\frac{\Theta_{}}{\Theta_{\infty }}\right)}^{3}$$

In this case, one can also formulate an analytical expression fitting well the exact numerical data for the entire coverage range [99]

(25) $$B(\Theta )=\left[1+0.812\bar{\Theta }+0.4258{\left(\bar{\Theta }\right)}^{2}+0.0716{\left(\bar{\Theta }\right)}^{3}\right]{\left(1-\bar{\Theta }\right)}^{3}$$

where $\bar{\Theta }=\frac{\Theta }{\Theta_{\infty }}$.

It was shown in Ref. [89] that the above results pertinent to hard particles can also be extended to the case of particles interacting via the short-range Yukawa potential. For the electrostatic double-layer interactions, the effective range of this potential is given by

(26) $$h^{*}=\frac{1}{\kappa d_{p}}\left[ln\frac{\phi_{o}}{\phi_{ch}}-ln\left(1+\frac{1}{\kappa d_{p}}ln\frac{\phi_{o}}{\phi_{ch}}\right)\right]$$

where $d_{p}$ is the particle size,

(27) $$\kappa^{-1}={\left(\frac{\varepsilon \;k\;T}{2\;e^{2}\;I}\right)}^{1/2}$$

is the Debye screening length, ε is the electric permittivity, *e* is the elementary charge, *I* is the ionic strength of the electrolyte solution, $\phi_{o}$ is electrostatic energy of two particles at close separations, and $\phi_{ch}$ is the characteristic interaction energy of the order of one *kT* unit.

Consequently, one can calculate the jamming coverage for interacting particles (referred to as the maximum coverage) from the relationship

(28) $$\Theta_{mx}=\;\Theta_{\infty }\frac{1}{{\left(1+h^{*}\right)}^{2}}$$

Knowing $\Theta_{mx}$*,* one can use Equation (25) to calculate the blocking function substituting $\bar{\Theta }=\frac{\Theta }{\Theta_{mx}}$.

Analogous solutions describing the blocking effects and the maximum coverages for anisotropic particles are given in Ref. [89].

## 3. Modeling QCM Response—Heterogeneous (Particle-Like) Load

### 3.1. General Considerations

The theoretical foundations of the QCM method have been extensively discussed in the literature [48,59,83,84]. In this work, we only present basic concepts, with attention focused on recent theoretical results pertinent to the heterogeneous load of the sensor. It is worth emphasizing that the load for nano- and microparticles is typically equal to 20–200 mg m^−2^ (Ref. [48]), which is considerably lower than the quartz crystal surface density, which is equal to ca. 5 × 10^6^ mg m^−2^ (for the crystal thickness of 0.2 cm). Therefore, one can assume that the particle deposition occurs under the small-load regime [101], where the complex QCM response is given by [48]

(29) $$\Delta f^{*}=\Delta f+\frac{f_{F}}{2n_{0}}\Delta D_{}^{}i$$

where Δf is the frequency shift, $f_{F}$ is the fundamental frequency of the sensor oscillations, $n_{0}$ is the overtone number, ΔD is the dissipation shift, and *i* is the imaginary number.

The frequency and dissipation shifts are explicitly given by [48]

(30) Δf=−fFπZqIm(ΔZL*)ΔD=2n0πZqRe(ΔZL*)

where $\Delta Z_{L}^{*}$ is the complex load impedance expressed relative to a reference state and $Z_{q}$ is the acoustic impedance of the quartz sensor equal to 8.8 × 10^6^ kg m^−2^ s^−1^.

$\Delta Z_{L}^{*}$ is defined as the ratio of the tangential stress (force per unit area) to the crystal surface velocity

(31) $$\Delta Z_{L}^{*}=\frac{\sigma^{*}}{V_{i}^{*}}=\frac{F_{i}^{*}}{S_{i}V_{i}^{*}}$$

where $\sigma^{*}$ is the complex tangential stress, $V_{i}^{*}$ is the complex sensor tangential velocity, $F_{i}^{*}$ is the tangential force acting on the crystal, and $S_{i}$ is the surface area.

### 3.2. The Rigid Contact Regime

The oscillatory motion of the QCM sensor creates in the adjacent fluid a standing wave propagating in the direction perpendicular to its surface. The wave amplitude exponentially decays with the distance from the sensor, inversely proportionally to the penetration depth,δ, also referred to as the hydrodynamic boundary layer, whose thickness is given by the formula [48]

(32) δ=(2vω)1/2=(ηπfFn0ρf)12

where v is the fluid kinematic viscosity, $\omega =2\pi f_{F}n_{0}$ is the sensor oscillation angular velocity, η is the fluid dynamic viscosity, and $\rho_{f}$ is the fluid density.

For particle suspensions in aqueous solutions at the temperature of 298 K, where the kinematic viscosity is equal to 0.00893 cm^2^ s^−1^ and for the fundamental frequency of 5 × 10^6^ s^−1^ (Hz), δ is equal to 238 and 71.6 nm for the first and the 11th overtone, respectively. For distances considerably larger than δ, the fluid motion vanishes creating a stagnant core volume. Therefore, particles of a size considerably exceeding δ, for example polymer microspheres, bacteria and cells attached to sensors are effectively immersed in a stagnant fluid.

On the other hand, for distances much smaller than δ, the hydrodynamic flow can be approximated as an oscillating but quasi-stationary simple shear flow [38] whose rate only depends on the angular velocity of the sensor, i.e., the overtone number and its amplitude. This significantly simplifies the analysis of the QCM deposition kinetics of particles of a size much smaller than δ, i.e., most protein molecules, some viruses and nanoparticles.

The shearing flow exerts an oscillatory hydrodynamic force on the sensor surface and on the attached particles. For firmly fixed particles, which cannot translate nor rotate, the hydrodynamic force on the particles is equal to the force on the sensor with a negative sign and can be, therefore, treated as the excess resistance force. Analogously, for the rigid contact regime, the inertia force on particles that is proportional to its mass and the sensor acceleration, is fully transferred to the sensor. This effect physically corresponds to the increase of the sensor mass by an increment equal to the particle mass, which corresponds to the Sauerbrey regime [83,101].

Consequently, under the rigid contact regime, the net force exerted on the sensor consists of the hydrodynamic and the inertia components [50]

(33) $$\Delta F^{*}=N_{p}\left(\Delta F_{h}^{*}+V_{i}{}^{*}\omega \;m_{p}i\right)$$

where $\Delta F^{*}$ is the net force on the sensor (a complex quantity), *N_p_* is the number of particles attached to the sensor and $\Delta F_{h}{}^{*}$ is the excess hydrodynamic force on the sensor induced by one particle that generally depends on the particle coverage and the structure of the particle layer [102].

Using Equation (31), one can express the mechanical impedance of the sensor in the following form

(34) $$\Delta Z_{L}^{*}=\omega \;m_{p}N\left({\bar{\Delta F}}_{h}^{*}+i\right)$$

where

(35) $${\bar{\Delta F}}_{h}^{*}=\frac{\Delta F_{h}^{*}}{V_{i}^{*}\omega \;m_{p}}$$

is the scaled excess hydrodynamic force and $N=N_{p}/S_{i}$ is the surface concentration of particles defined above.

Considering Equations (30) and (34), the frequency and dissipation shifts are given by

(36) Δf=−fFω mpπZqNIm(ΔZL*)ΔD=2ω mpn0πZqNRe(ΔFh)¯*

It is also useful to define the commonly used variable

(37) ΔD¯=−ΔD/(Δf/n0)=2fFRe(ΔF¯h*)Im(ΔF¯h*+i)

**Figure 1:**
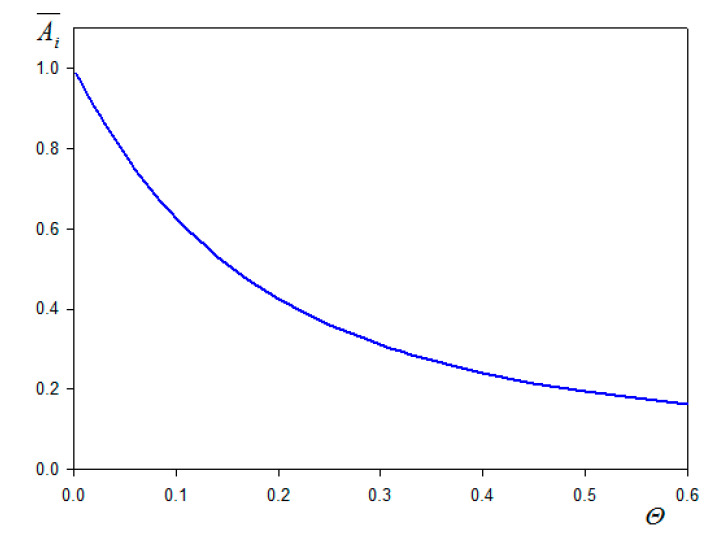
The $\bar{A_{i}}(\Theta )$ function for spherical particles.

One should highlight that Equations (34)–(37) are valid for arbitrary particle sizes and shapes. However, an explicit calculation of the frequency and dissipation shifts requires calculations of the complex hydrodynamic force on the attached particles as a function of their coverage. The force has been recently calculated for spherical and spheroidal particles applying finite element modeling and using the Lattice Boltzmann approximation [44,45]. On the other hand, in Ref. [50], the complex force on spherical particles attached to the sensor was calculated assuming a quasi-stationary oscillating flow model and can be expressed as

(38) $${\bar{\Delta F}}_{h}^{*}=F_{1}(\bar{\delta })\bar{A_{i}}(\Theta )+F_{2}(\bar{\delta })\bar{A_{i}}(\Theta )i$$

with

(39) $$F_{1}(\bar{\delta })=\frac{3}{8}C_{i}^{o}\frac{\rho_{f}}{\rho_{p}}\left(\cos\frac{1}{\bar{\delta }}-\sin\frac{1}{\bar{\delta }}\right)e_{}^{-1/{\bar{\delta }}_{}^{}}\bar{\delta }\;F_{2}(\bar{\delta })=\frac{3}{8}C_{i}^{o}\frac{\rho_{f}}{\rho_{p}}\left(\cos\frac{1}{\bar{\delta }}+\sin\frac{1}{\bar{\delta }}\right)e_{}^{-1/{\bar{\delta }}_{}^{}}\bar{\delta }$$

where for spherical particles $C_{i}^{o}$ = 10.21, $\bar{\delta }=\delta /a$ is the dimensionless parameter characterizing the ratio of the hydrodynamic boundary layer thickness to the particle dimension (radius), and $\bar{A_{i}}(\Theta )$ is the universal function (shown in Figure 1) describing the hydrodynamic flow damping in the particle layer attached to the sensor. It is given by the interpolation formula [102]

(40) $$\bar{A_{i}}(\Theta )=\frac{1-e^{-C_{i}^{o}\Theta }}{C_{i}^{o}\Theta }$$

where the coverage θ is calculated for spherical particles from the formula

(41) $$\Theta =\pi a^{2}N=\frac{3}{4a\rho_{p}}\Gamma$$

It is also shown in Ref. [101] that the $\bar{A_{i}}(\Theta )$ function, which also describes the effect of the streaming current reduction, is independent for spherical particles on the structure of their layers.

However, it should be mentioned that Equation (39) becomes less accurate for $\bar{\delta }$ < 2 where the hydrodynamic boundary layer thickness becomes smaller than the particle diameter. In this case, the following analytical solutions were derived in Ref. [46] describing the $F_{1}(\bar{\delta })$ and $F_{2}(\bar{\delta })$ functions

(42) $$F_{1}(\bar{\delta })=\frac{\rho_{f}}{\rho_{p}}\left(1+\frac{9}{4}\bar{\delta }\right)\bar{\delta }\;F_{2}(\bar{\delta })=1+\frac{\rho_{f}}{\rho_{p}}\left[\frac{1}{2}+\frac{9}{4}\bar{\delta }\right]$$

It should be mentioned, however, that in deriving Equation (42), the hydrodynamic interactions among particles were not considered; hence, the $\bar{A_{i}}(\Theta )$ function was assumed to be equal to unity. If one considers that under this regime the hydrodynamic boundary thickness is smaller than the particle dimensions, one can expect that this assumption is valid for particle coverage up to $\Theta_{mx}/{\left(1+\bar{\delta }\right)}^{2}$, where $\Theta_{mx}$ is the maximum coverage equal to 0.547 for spherical particles under the random sequential adsorption regime (RSA) [100].

**Figure 2:**
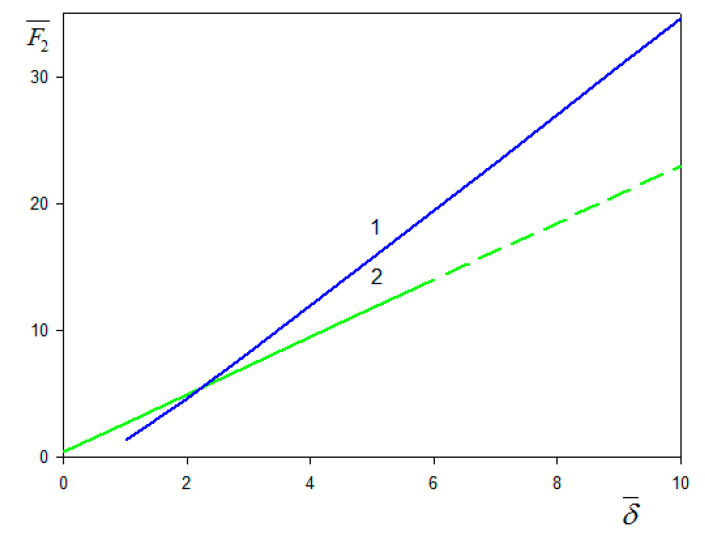
The $F_{2}(\bar{\delta })$ function calculated from Equation (39) (blue line number 1) and from Equation (42), (green line 2), $\frac{\rho_{f}}{\rho_{p}}$ = 1.

Because the $F_{2}(\bar{\delta })$ function, shown in Figure 2, comprises no adjustable parameters, it is valid for spherical particle of arbitrary size and for all overtone numbers.

Considering Equation (36), one can explicitly express the frequency and the dissipation shifts in the following form:

(43) $$\Delta f=-\frac{f_{F}\omega \;m_{p}}{\pi Z_{q}}N\left[1+F_{2}(\bar{\delta })\bar{A_{i}}(\Theta )\right]$$

(44) $$\Delta D=\frac{2\omega \;m_{p}}{n_{0}\pi Z_{q}}NF_{1}(\bar{\delta })\bar{A_{i}}(\Theta )$$

Accordingly, the $-\Delta D/\left(\Delta f/n_{0}\right)$ parameter is given by

(45) $$-\Delta D/\left(\Delta f/n_{0}\right)=\frac{2}{f_{F}}F_{1}(\bar{\delta })\;\bar{A_{i}}(\Theta )/\left(1+F_{2}(\bar{\delta })\bar{A_{i}}(\Theta )\right)$$

For $\bar{\delta }$ >> 1, i.e., for the nanoparticle size range and lower overtone number, Equation (43) with $\bar{A_{i}}(\Theta )$ calculated from Equation (40) assumes the dimensional form

(46) −Δf/n0=Γ/Cs+(ηρfπfF)12n0−1/2(1−e−Cio34aρpΓ)/Cs

where $C_{s}=\frac{Z_{q}}{2f_{F}^{2}\;}$ is the Sauerbrey constant, which is equal to 0.177 (mg m^−2^ s) for $f_{F}=\;$5 × 10^6^ Hz.

The first term in Equation (46) describes the inertia contribution, which is independent of the overtone number, and the second term characterizes the hydrodynamic force contribution, which decreases as $n_{0}^{-1/2}$. It should be mentioned that because of the exponential term, the inversion of Equation (46) to obtain the dry mass of adsorbed solute Γ using the measured $-\Delta f/n_{0}$ is only feasible using numerical methods.

Equation (46) can also be expressed in the simpler form

(47) ΓQ=Γ+(ηρfπfF)12n0−1/2(1−e−Cio34aρpΓ)

where the quantity

(48) $$\Gamma_{Q}=C_{s}(-\Delta f/n_{0})$$

can be treated as the QCM wet mass.

Analogously, the dissipation shift and the parameter $-\Delta D/\left(\Delta f/n_{0}\right)$ are given by

(49) ΔD=2fFCs(ηρfπfF)12n0−1/2(1−e−Cio34aρpΓ)=CDn0−1/2(1−e−Cio34aρpΓ)

(50) −ΔD/(Δf/n0)=2fF(ηρfπfF)12n0−1/2(1−e−Cio34aρpΓ)/(1+(ηρfπfF)12n0−1/2(1−e−Cio34aρpΓ))

where CD=2fFCs(ηρfπfF)12

Equation (49) can be analytically inverted, which yields the following expression for the dry mass

(51) $$\Gamma =-\frac{4a\rho_{p}}{3_{F}C_{i}^{o}}{\left[\ln(1-\Delta D_{}^{}n_{0}^{1/2}/C_{D})\right]}^{-1}$$

For a lower coverage range where $C_{i}^{o}\frac{3}{4a\rho_{p}}\Gamma <<1$, the expression for $-\Delta f/n_{0}$, Equation (46) simplifies to the linear form

(52) −Δf/n0=[1+3Cio4aρp(ηρfπfF)12n0−1/2]Γ/Cs

Thus, in this case, the QCM measurements allow for an explicit determination of the dry coverage of particles from the formula

(53) Γ=−Cs(Δf/n0)/[1+3Cio4aρp(ηρfπfF)12n0−1/2]=ΓQ/[1+3Cio4aρp(ηρfπfF)12n0−1/2]

On the other hand, the quantity $\Gamma_{Q}/\Gamma_{}$, which is defined as the water factor [38], is explicitly given by

(54) ΓQ/Γ=w=1+3Cio4aρp(ηρfπfF)12n0−1/2

Equations (46), (51) and (53) indicate that under the rigid contact regime, the dry coverage of attached particles can be ab initio calculated using the normalized frequency and dissipation shifts derived from QCM measurements.

### 3.3. The Soft Contact Regime

If the adhesion strength between the sensor and the attached particles is not adequate to ensure its full immobilization, the situation becomes more complex as discussed in Refs. [38,48]. Under this regime, the particles can undergo lateral and angular displacements and they can even translate over the sensor under the lubrication motion regime. The magnitude of these movements is dependent on several parameters characterizing the particle/sensor surface interactions such as their size and shape, the Hamaker constant, ionic strength, pH, zeta potential, and elasticity (Young) moduli [48]. Additionally, the heterogeneity of the sensor and its topography, especially the roughness, which controls the adhesion force strength, play significant roles.

One of few theoretical models that take into account the most important effects was formulated in Ref. [48], assuming that the attached particles execute a damped oscillatory motion without undergoing translational motion. The lateral and angular displacements of particles are governed by the complex spring constant $\kappa_{c}^{*}$ given by

(55) $$\kappa_{c}^{*}=\frac{\kappa_{s}^{*}\kappa_{b}^{*}}{\kappa_{s}^{*}+\kappa_{b}^{*}}=\kappa_{c}^{}+\omega \xi i=\kappa_{c}^{}(1+\omega \bar{\xi }i)$$

where $\kappa_{s}^{*}$ is the shearing (complex) spring constant, $\kappa_{b}^{*}$ is the complex bending spring constant, $\kappa_{c}^{}$ is the real component of the spring constant, ξ is the damping coefficient, and $\bar{\xi }=\xi /\kappa_{c}^{}$.

It is also shown in Ref. [81], considering the Johnson–Kendall–Roberts (JKR) theory, that the real part of the bending constant can be calculated for spherical particles from the formula

(56) $$\kappa_{c}≅\kappa_{b}=6\pi W_{A}$$

where *W_A_* is the work of adhesion per unit area governed by the van der Waals and electrostatic interactions, which can be calculated from the formula [89]

(57) $$W_{A}=\frac{A_{123}}{12h_{m}^{2}}+kT\kappa^{-1}I_{}Y_{i}Y_{p}{}_{}e^{-\kappa^{}h_{m}}$$

where *A*_123_ is the Hamaker constant for the interactions of the particle with the interface through the solvent (electrolyte), $h_{m}=\delta_{m}$ is the minimum approach distance of the particle to the substrate.

(58) $$Y_{i}=4_{}\tan h\left(\frac{\zeta_{i}e}{4kT}\right)\;Y_{p}=4_{}\tan h\left(\frac{\zeta_{p}e}{4kT}\right)$$

and $\zeta_{i}$, $\zeta_{p}$ are the zeta potentials of the interface and the particle.

Equation (57) indicates that the work of adhesion depends on the minimum approach distance of the particle, which can be approximated as the average roughness size of the sensor. Therefore, for sensors with the rms factor exceeding 1 nm, the work of adhesion significantly decreases, which may result in a transition from the rigid to the soft contact regime.

Particle displacements governed by $\kappa_{c}^{*}$ generate a damped elastic force, which is shifted in phase compared to the sensor velocity. Considering this effect, and using Equation (55), the following expression for the complex mechanical impedance was derived [48,50]

(59) $$\Delta Z_{L}^{*}=\omega \;m_{p}N\frac{{\bar{\Delta F}}_{t}^{}}{1+c_{f}\frac{\omega^{2}m_{p}}{\kappa_{c}(\omega_{}^{2}{\bar{\xi }}^{2}+1)}(\omega {\bar{\xi }}_{}+i){\bar{\Delta F}}_{t}^{}}=\omega \;m_{p}N\frac{{\bar{\Delta F}}_{t}^{}}{1+C(\omega {\bar{\xi }}_{}+i){\bar{\Delta F}}_{t}^{}}$$

where, as previously defined

(60) $${\bar{\Delta F}}_{t}^{}=F_{1}(\bar{\delta })\bar{A_{i}}(\Theta )+F_{2}(\bar{\delta })\bar{A_{i}}(\Theta )i=F_{1}^{\prime }+F_{2}^{\prime }i$$

is the normalized translational hydrodynamic force on particles and $c_{f}≅1-{\bar{\Delta F}}_{r}^{}/{\bar{\Delta F}}_{t}^{}$ is the ratio of the rotational $\left({\bar{\Delta F}}_{r}^{}\right)$ to the translation hydrodynamic forces, assumed to be a real number in the order of unity and independent of the $\bar{\delta }$ parameter. The dimensionless constant C in Equation (59) is given by

(61) $$C=c_{f}\frac{\omega^{2}\;m_{p}}{\kappa_{c}{\left(\omega_{}^{2}{\bar{\xi }}^{2}+1\right)}^{}}$$

Equation (61) indicates that the *C* constant increases with the particle mass, i.e., proportionally to the cube of their size, and inversely proportionally to the spring constant, which means that it can attain large values for microparticle size and for low work of adhesion. Consequently, for larger values of *C*, if the term Re [$C(\omega {\bar{\xi }}_{}+i){\bar{\Delta F}}_{t}^{}$]>> 1, and Equation (59) simplifies to the limiting

(62) $$\Delta Z_{L}^{*}=\omega \;m_{p}N\frac{1}{C{\left(\omega_{}^{2}{\bar{\xi }}^{2}+1\right)}_{}}(\omega \bar{\xi }-i)=\frac{\kappa_{c}}{c_{f}\omega }N(\omega \bar{\xi }-i)$$

Combining Equations (36), (59) and (60), one obtains the general expressions for the frequency and dissipation shifts

(63) $$C_{s}(-\Delta f/n_{0})=\Gamma_{Q}=\Gamma \frac{F_{2}^{\prime }-C\left(F_{1}^{\prime }{}^{2}+F_{2}^{\prime }{}^{2}\right)}{{\left[1+C(F_{1}^{\prime }\omega \bar{\xi }-F_{2}^{\prime })\right]}^{2}+C^{2}{\left(F_{1}^{\prime }+F_{2}^{\prime }\omega \bar{\xi }\right)}^{2}}$$

(64) $$\Delta D=\frac{2}{f_{F}C_{s}}\Gamma \frac{F_{1}^{\prime }+C_{}\omega \bar{\xi }\left(F_{1}^{\prime }{}^{2}+F_{2}^{\prime }{}^{2}\right)}{{\left[1+C(F_{1}^{\prime }\omega \bar{\xi }-F_{2}^{\prime })\right]}^{2}+C^{2}{\left(F_{1}^{\prime }+F_{2}^{\prime }\omega \bar{\xi }\right)}^{2}}$$

From Equation (63) one can infer that if $C_{}<<\frac{F_{2}^{\prime }}{\left(F_{1}^{\prime }{}^{2}+F_{2}^{\prime }{}^{2}\right)}$ the influence of the particle oscillatory motion becomes negligible; hence one recovers the rigid contact regime. This criterion can be expressed using Equations (38) and (39) for $\bar{\delta }$ > 2 and $\omega \bar{\xi }$ < 1 in the following form

(65) $$\frac{3}{8}C_{i}^{o}\frac{\rho_{f}}{\rho_{p}}\bar{\delta }\frac{\omega^{2}\;m_{p}}{\kappa_{c}}<<1$$

It is predicted from this equation that the rigid contact regime is most likely for nanoparticles and for small overtone numbers provided that the adhesion strength is sufficient, i.e., for smooth surfaces characterized by the homogeneous distribution of surface properties.

On the other hand, Equation (63) indicates that the frequency shift becomes positive if $C_{}>\frac{F_{2}^{\prime }}{\left(F_{1}^{\prime }{}^{2}+F_{2}^{\prime }{}^{2}\right)}$. Hence, as pointed out in Ref. [81], for large overtone numbers and larger particles, one can expect positive frequency shifts, resulting in negative QCM mass.

## 4. Experimental Results

### 4.1. General Considerations

Because of its numerous advantages, the QCM method is used in an increasing number of investigations for nanoparticles involving liposomes and vesicles [44,45,56,96], macroions [31,51,52,53], proteins [54,55,56,57,58,59,60,61,62,63,64,65], viruses [56,66,67,68,69,70], bacteria [71,72,73,74,75,76,77] and living cells, comprising cancerous ones [48,78,79,80,81,82]. However, as mentioned above, a quantitative theoretical interpretation of QCM measurements for bioparticles is often not feasible because the frequency and the dissipation shifts depend on many inadequately controlled parameters. One should primarily mention the bioparticle shape, conformations and orientation on the sensor, surface properties (elasticity, charge distribution), sensor roughness, and its surface chemistry.

To experimentally determine the significance of these effects, one should acquire information about adsorption kinetics for the same solute/interface system using complementary experimental techniques. This was realized in several investigations applying ex situ experimental methods such as ellipsometry [26], SPR [31], OWLS [26,27,28], AFM and XPS [49,50,62]. The ratio of the wet to the dry coverage derived in this way can be used to calculate the experimental hydration functions and estimate the validity of theoretical approaches. In Refs. [56,57,59], more direct methods were developed to monitor the dry mass of adsorbed proteins in situ using reflectometry, ellipsometry and SPR measurements.

An alternative method was applied in Refs. [3,27,28,49,50,60,61,62], where the dry mass of adsorbed solute (proteins) was derived from the solution of the mass transfer equation discussed in the first section. The mass transfer rate constant necessary for performing such calculations can be derived either ab initio via the numerical solution of the Navier-Stokes equation [96] or from calibrating experiments involving metal nanoparticles characterized by a large density such as silver [42] and gold [3]. Except for yielding the mass transfer rates, results of such systematic QCM investigations of nanoparticle deposition kinetics in well-defined systems can be used as reference data for the interpretation of bioparticle adsorption mechanisms.

Given the significance of the experimental data acquired for nano- and microparticles, they are discussed first in this section. Then, some selected experimental data obtained for viruses and protein molecules are presented in the final section of this work.

For the interpretation of experimental data it is useful to define several functions used in the literature [49,56,59], primarily, the following one,

(66) $$w=\frac{\Gamma_{Q}}{\Gamma_{}}$$

As above mentioned, $\Gamma_{Q}$ is calculated from the dependence

(67) $$\Gamma_{Q}=-C_{s}{}_{}^{}\Delta f/n_{0}$$

Physically, the $\frac{\Gamma_{Q}}{\Gamma_{}}$ function referred to in the literature as the water factor *w* [49,59] represents the ratio of the total force to the inertia force exerted on the sensor due to adsorbed particles.

Equations (66) and (67) indicate that the calculation of the water factor requires, besides the QCM signal, information about the dry particle coverage, acquired by an independent experimental method or from theoretical modeling.

Another commonly used function, *H,* is defined as

(68) $$H=1-\frac{\Gamma }{\Gamma_{Q}}=1-1/w$$

It is also useful to define the following function characterizing solely the contribution of the hydrodynamic force [49,50]

(69) $$\bar{v}=\frac{\rho_{p}}{\rho_{f}}(w-1)$$

Exploiting the theoretical results discussed in the previous section, these functions can be expressed under the rigid contact regime in the following form

(70) $$w=1+F_{2}(\bar{\delta })\bar{A_{i}}(\Theta )$$

(71) $$H=\frac{F_{2}(\bar{\delta })\bar{A_{i}}(\Theta )}{1+F_{2}(\bar{\delta })\bar{A_{i}}(\Theta )}$$

(72) $$\bar{v}=\frac{\rho_{p}}{\rho_{f}}F_{2}(\bar{\delta })\bar{A_{i}}(\Theta )$$

For $\bar{\delta }=\delta /a>2$ one has

(73) $$F_{2}(\bar{\delta })=\frac{3}{8}C_{i}^{o}\frac{\rho_{f}}{\rho_{p}}\left(\cos\frac{1}{\bar{\delta }}+\sin\frac{1}{\bar{\delta }}\right)e_{}^{-1/{\bar{\delta }}_{}^{}}\bar{\delta }\;\bar{A_{i}}(\Theta )=\frac{1-e^{-C_{i}^{o}\Theta }}{C_{i}^{o}\Theta }$$

For $\bar{\delta }<2$ one has

(74) $$F_{2}(\bar{\delta })=\frac{\rho_{f}}{\rho_{p}}\left(\frac{1}{2}+\frac{9}{4}\bar{\delta }\right)\;\bar{A_{i}}(\Theta )=1$$

The scaled hydrodynamic boundary layer thickness is given by

(75) δ¯=(2vωa2)1/2=(ηπfFn0a2ρf)12

The solute coverage is defined as

(76) $$\Theta =S_{g}N=S_{g}\Gamma /m_{p}$$

where *S_g_* is the characteristic particle cross-section area equal to $\pi a^{2}$ for spherical particles. Thus, their coverage is given by

(77) $$\Theta =\pi a^{2}N=3\Gamma /4a\rho_{p}$$

### 4.2. Reference Results for Nanoparticles

**Figure 3:**
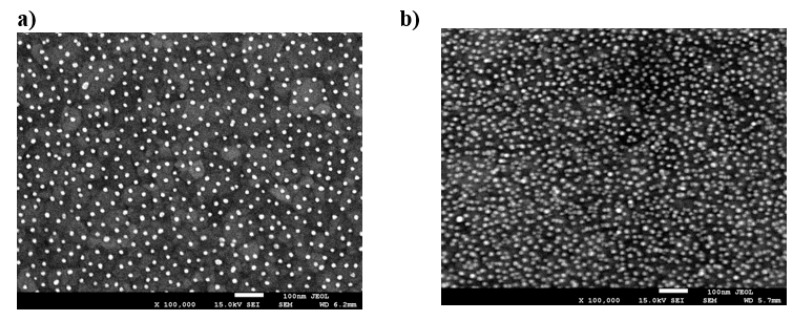
SEM micrographs of gold nanoparticle monolayers on a QCM gold sensor. (**a**) Ionic strength 10^−4^ M (Θ = 0.098), (**b**) ionic strength 10^−2^ M (Θ = 0.28). Reprinted (adapted) with permission from Ref. [3] Copyright © (2016) American Chemical Society.

In Refs. [3,5], deposition of gold nanoparticles, synthetized by the chemical reduction method on poly(allyl amine hydrochloride) (PAH)-modified gold sensors, was studied using the QCM-D method and ex situ electron microscope imaging. The particle size was equal to 14 ± 2 nm and their zeta potential varied between −58 and −47 mV for ionic strength of 10^−4^ and 10^−2^ M, respectively (at pH equal to 7.4). The density of the particles was equal to 19.3 g cm^−3^. The SEM micrographs of gold nanoparticle monolayers on a QCM gold sensor for different ionic strength regulated by NaCl addition are shown in Figure 3.

**Figure 4:**
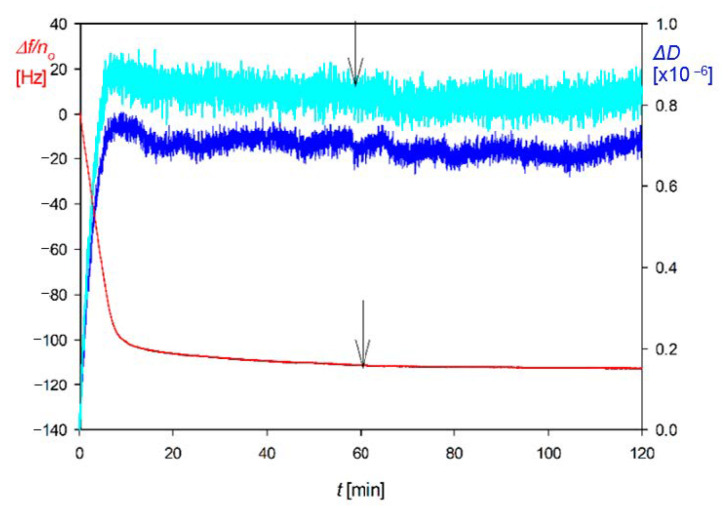
QCM measurements of gold nanoparticle deposition at the PAH-modified Au sensor expressed as the normalized frequency shift $\Delta f/n_{0}$ [Hz] and the dissipation shift ΔD (right hand axis) vs. the time for the 3rd and 11th overtone, pH 7.4 (PBS buffer), bulk suspension concentration 30 mg L^−1^, ionic strength 10^−4^ M, flow rate *Q* = 1.33 × 10^−3^ cm^3^ s^−1^. The arrows show the beginning of the rinsing run. Reprinted (adapted) with permission from Refs. [3,5]. Copyright © (2016) American Chemical Society.

A typical QCM kinetic run acquired at pH 7.4 (fixed by a PBS buffer), for bulk suspension concentration 30 mg L^−1^ and ionic strength 10^−4^ M, is shown in Figure 4 as the dependence of the normalized frequency and dissipation shifts on the deposition time.

One can observe that the frequency shift induced by particle deposition is practically the same for the 3rd and 11th overtone. This behavior is in agreement with Equation (39), formulated in the previous section, which predicted that for large particle density, the $F_{2}(\bar{\delta })$ function describing the relative significance of the hydrodynamic force would become smaller than unity.

Therefore, in this case, a dominant role is played by the term describing the inertia force, which is independent of the overtone number. It is also seen in Figure 4 that the frequency change during the desorption run, where pure electrolyte was flushed through the cell, was negligible.

**Figure 5:**
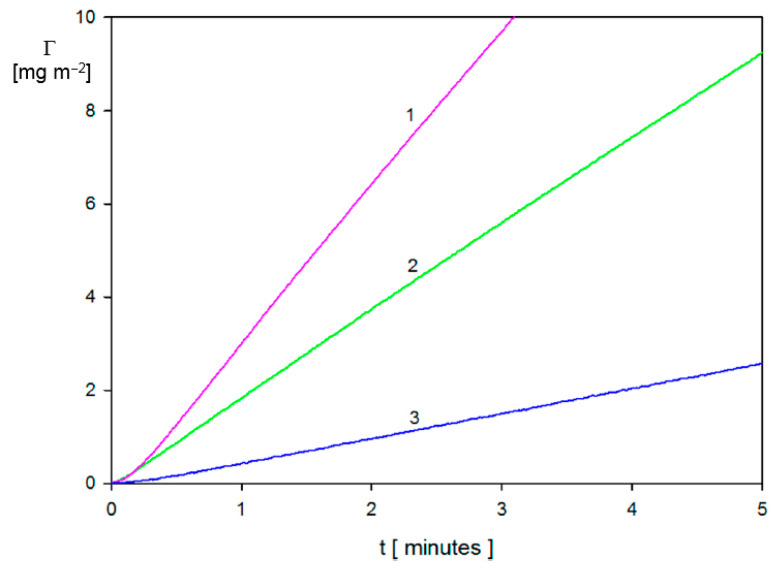
The kinetics of gold nanoparticle deposition on PAH-modified Au sensor determined by QCM for various bulk suspension concentrations: (1) 50 mg L^−1^, (2) 30 mg L^−1^, (3) 10 mg L^−1^; pH 7.4 (PBS); *I* = 10^−2^ M, *Q* =1.33 × 10^−3^ cm^3^s^−1^. Reprinted (adapted) with permission from Ref. [3] Copyright © (2016) American Chemical Society.

To express the particle deposition kinetics in terms of the mass coverage, Equation (67) was applied in Ref. [3]. Typical short-time kinetic runs obtained in this way for various bulk suspension concentration equal to 10, 30 and 50 mg L^−1^ are shown in Figure 5. As can be seen, the duration of the transient deposition regime that is characterized by the non-linear dependence of *Γ* on *t* was approximately 10 s, which is considerably smaller than the duration of the entire deposition/desorption run, typically lasting 200 min. Hence, for deposition times exceeding 10 s, the particle coverage linearly increases for all bulk suspension concentrations, which indicates that the deposition rate is controlled by bulk transport with negligible influence of surface blocking effects. The linearity of the kinetic runs also confirms that the hydrodynamic force term, which is dependent on the particle coverage, i.e., the deposition time, played a negligible role. An analogous behavior was experimentally observed in Ref. [42] for silver particle deposition on PAH-modified gold sensors.

**Figure 6:**
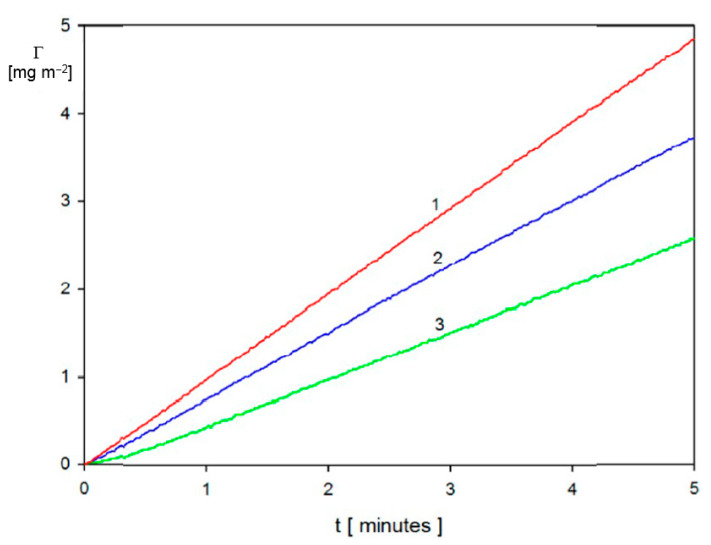
The kinetics of gold nanoparticle deposition on PAH-modified Au sensor (pH 7.4, PBS buffer), concentration of gold nanoparticles equal to10 mg L^−1^, *I* = 10^−2^ M determined by QCM for various volumetric flow rates *Q*: (1) 6.16 × 10^−3^ cm^3^ s^−1^, (2) 2.5 × 10^−3^ cm^3^ s^−1^, (3) 1.33 × 10^−3^ cm^3^ s^−1^. Reprinted (adapted) with permission from Ref. [3] Copyright © (2016) American Chemical Society.

To determine the dependence of the mass transfer rate constants on the volumetric flow rate of the suspension, a series of kinetic experiments was carried out in Ref. [3]. The short-time kinetics of gold particle deposition obtained for bulk suspension concentration of 10 mg L^−1^ and various flow rates is shown in Figure 6.

These results and others acquired in Ref. [42] for various bulk suspension concentrations or ionic strength confirmed that the mass transfer rate constant governing the particle deposition under the bulk-controlled regime can be expressed as

(78) $$k_{c}=C_{f}Q^{\frac{1}{3}}D^{\frac{2}{3}}$$

with the $C_{f}$ equal to 1.9 cm^−4/3^. It is also confirmed in Ref. [3] that the $C_{f}$ constant was practically independent of the ionic strength and pH of the suspension.

Considering Equation (78), one can calculate the dry coverage of any solute, e.g., protein molecules for the QCM cell under the linear adsorption regime from the dependence

(79) $$\Gamma =10^{}C_{f}Q^{\frac{1}{3}}D^{\frac{2}{3}}c_{b}t$$

where Γ is expressed in mg m^−2^ and $c_{b}$ in mg L^−1^.

**Figure 7:**
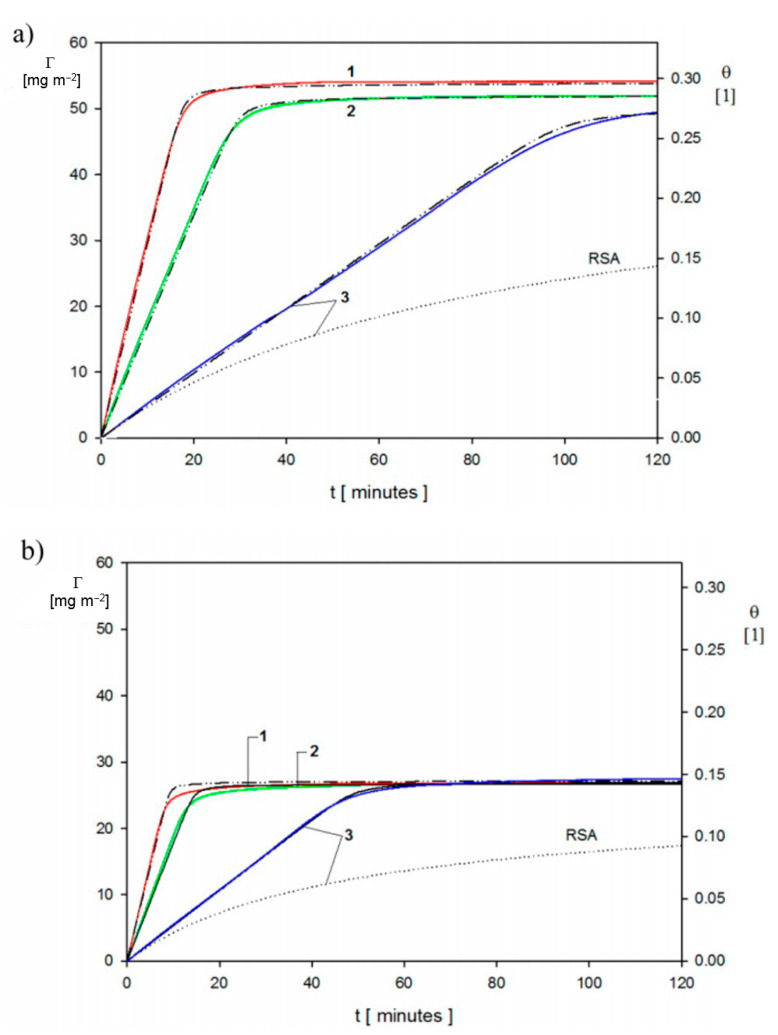
The kinetics of gold nanoparticle deposition on PAH-modified Au sensor determined by QCM-D for bulk suspension concentration of (1) 50 mg L^−1^, (2) 30 mg L^−1^, (3) 10 mg L^−1^; pH 7.4 (PBS buffer), *Q* = 2.5 × 10^−3^ cm^3^ s^−1^. (**a**) *I* = 10^−2^ M, (**b**) *I* = 10^−3^ M. The dashed-dotted lines show the theoretical results derived from the general RSA model, and the dotted lines show the results calculated using the standard RSA model. Reprinted (adapted) with permission from Ref. [3] Copyright © (2016) American Chemical Society.

The mass transfer rate is used as the primary input parameter needed in the theoretical modeling of particle deposition kinetics for the large coverage regime, where the blocking effects begin to play a decisive role. Kinetic runs pertinent to this regime, which were obtained in Ref. [3] for ionic strength of 10^−2^ M and 10^−3^ M (pH 7.4) and various bulk suspension concentrations, are shown in Figure 7. One can observe that for longer deposition times, a plateau coverage $\Gamma_{\mathrm{mx}}$ of the gold nanoparticle is attained independently of the bulk suspension concentration. This behavior was interpreted as an indication of an irreversible adsorption of particles. It is also interesting to mention that $\Gamma_{\mathrm{mx}}$ abruptly increases with ionic strength, which controls the electric double-layer thickness. This confirms an essential role of the lateral electrostatic interactions among deposited particles. 

These QCM results were calibrated using the ex situ SEM imaging of particle monolayers (see Figure 3) yielding the surface concentration of particles and, consequently, their absolute coverage from Equation (77). A satisfactory agreement between the two experimental methods was observed for various ionic strengths. One can also see in Figure 7 that the theoretical results derived from the general RSA model where the coupling of the bulk and surface transport is considered adequately reflect the experimental data for the entire range of bulk suspension concentration and deposition time. In contrast, the results calculated using the standard RSA model where the bulk transport resistance is neglected (shown in Figure 7 as dotted lines) significantly underestimate the experimental data.

Analogous QCM measurements of deposition kinetics are reported in Refs. [39,44,45] for nanoparticles characterized by a smaller density, where the hydrodynamic force effects governing the overtone dependence of the frequency shift, play a more significant role.

**Figure 8:**
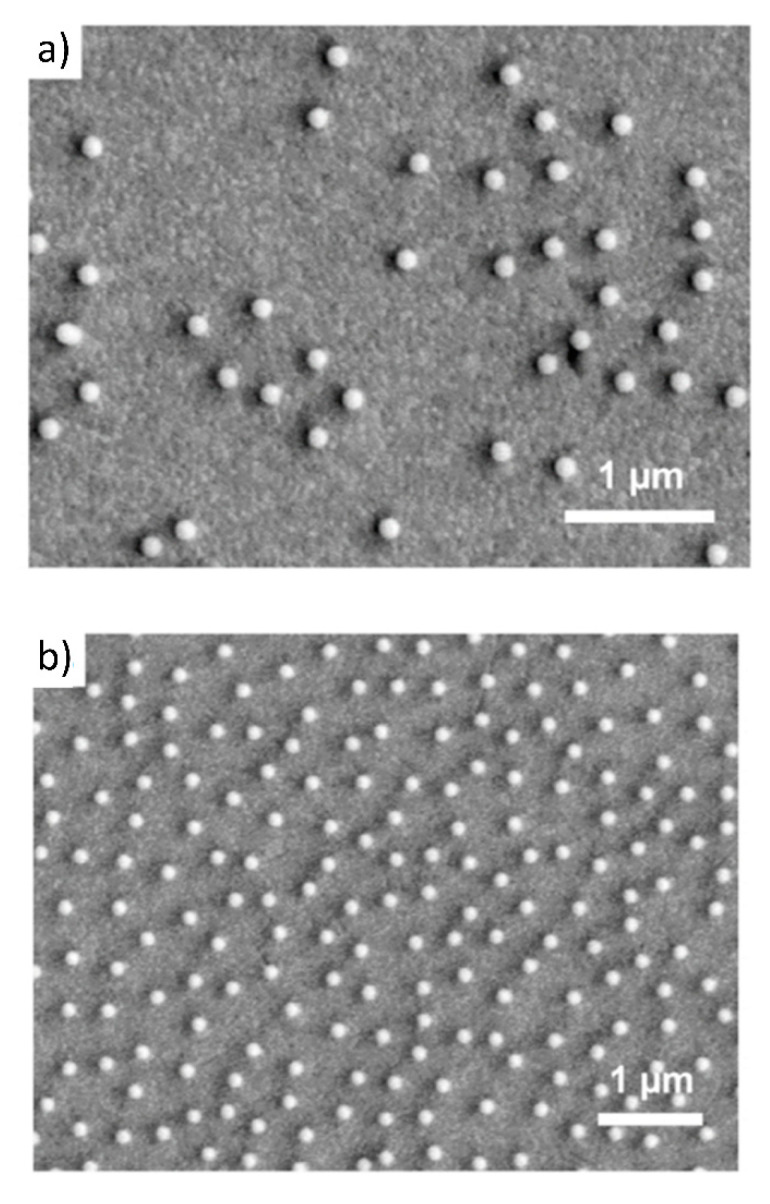
SEM images of positively charged AHAPS functionalized silica particles with a diameter of 137 ± 4 nm on a gold QCM sensor. The surface concentration of particles is equal to (**a**) 5.9 and (**b**) 2.2 μm^−2^. Reprinted (adapted) with permission from Ref. [41]. Copyright © (2015) American Chemical Society.

In Ref. [41], the deposition of silane (AHPS) functionalized silica particles of the size of 137 nm from ethanol suspensions on a gold sensor was investigated by QCM-D. Similarly, as in Ref. [3], the absolute particle coverage was quantitatively determined by ex situ SEM enumeration of particles (the particle layers are shown in Figure 8).

**Figure 9:**
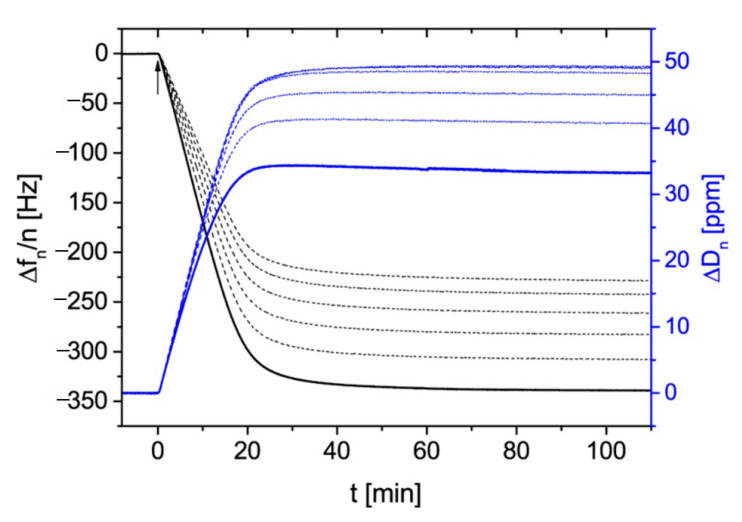
QCM-D measurement of AHAPS functionalized silica particles were deposited on a gold sensor, *Q* = 1.67 × 10^−3^ cm^3^ s^−1^. Black lines: normalized frequency changes for various overtones 3rd-13th; blue lines correspond to changes of dissipation for various overtones (3rd-13th). The bold solid lines correspond to the 3rd overtone. Reprinted (adapted) with permission from Ref. [41] Copyright © (2015) American Chemical Society.

The kinetic run acquired for the flow rate of 1.67 × 10^−3^ cm^3^ s^−1^ is shown in Figure 9 as the dependence of the normalized frequency and dissipation shifts on the deposition time. In contrast to the deposition of gold particles shown in Figure 4, there appear significant differences in the frequency and dissipation shifts among various overtones. Generally, the negative value of the frequency shift systematically increases with the overtone number, whereas the dissipation shift decreases with the overtone number. This behavior qualitatively agrees with what was theoretically predicted by Equations (46) and (49), which suggests that the experiments were performed under the rigid contact regime.

**Figure 10:**
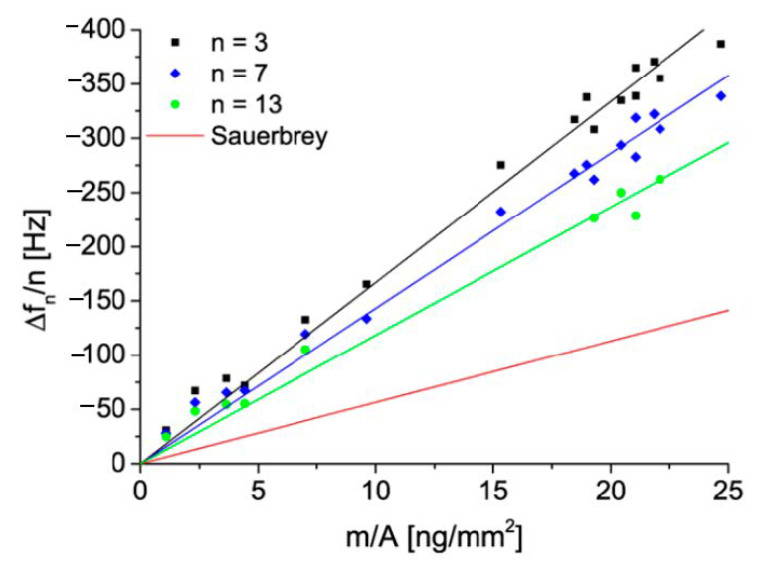
The normalized frequency change as a function of the surface mass density (coverage) determined from the SEM images for three overtones (black square—3rd overtone; blue diamond—7th overtone; green circle—9th overtone). Red line represents frequency changes based on the Sauerbrey equation. Reprinted (adapted) with permission from Ref. [41] Copyright © (2015) American Chemical Society.

A quantitative comparison with the theoretical model is also feasible because the QCM data acquired in Ref. [41] were expressed in terms of the mass concentration of particles derived from SEM images. In Figure 10, the dependence of the frequency shift on this parameter is shown for various overtones. The maximum coverage of 25 ng mm^−2^ (equal to 25 mg m^−2^) corresponds to the surface concentration of 9.5 μm^−2^ and the absolute particle coverage of *θ* = 0.14. The experimental frequency shifts obtained for various overtones as a function of the SEM dry coverage (black, blue and green lines) are compared with the frequency shift calculated from Equation (67) (red line in Figure 10). This allows the calculation of the water factor as the ratio of these frequency shifts. In this way, one obtains the experimental values of 3.3 and 2.2 for the 3rd and 13th overtones, which correspond to values of the hydration function *H* equal to 0.7 and 0.52, respectively. Consequently, the $\bar{v}=\frac{\rho_{p}}{\rho_{f}}(w-1)$ function describing the hydrodynamic force is equal to 6.1 and 2.8 for the 3rd and 13th overtone, respectively (taking the experimental values of $\rho_{p}$ = 1.93 g cm^−3^ and $\rho_{f}$ = 0.76 g cm^−3^). The corresponding theoretical values of this function calculated from Equations (72) and (73) for the 3rd overtone (where $\bar{\delta }=\delta /a$ = 2), and Equations (72) and (74) for the 13th overtone (where $\bar{\delta }$= 0.9) are 6.2 and 2.6, respectively, are in agreement with the experimental data. Therefore, the rigid contact regime was probable in the Grunwald et al. experiments [41].

Interesting experimental investigations of liposome deposition of a size equal to 68 nm on a titanium oxide QCM sensor showing an analogous behavior were reported in Refs. [44,45] and theoretically interpreted in terms of lattice Boltzmann numerical modeling.

On the other hand, in Ref. [50], systematic investigations of amidine nano- and microparticles deposition kinetics from aqueous solutions on the Si/SiO_2_ QCM sensor were carried out. The particle sizes derived from dynamic light scattering were equal to 67, 140, 360 and 810 nm (hereafter denoted as A70 and A140, A350, A800). All particles exhibited a large and positive zeta potential varying between 74 and 97 mV (at pH 4.0 and ionic strength of 0.01–0.001 M), which facilitated their irreversible deposition on the Si/SiO_2_ QCM sensor, the zeta potentials of which at pH 4.0 were equal to −20 and −32 mV for the same ionic strengths.

**Figure 11:**
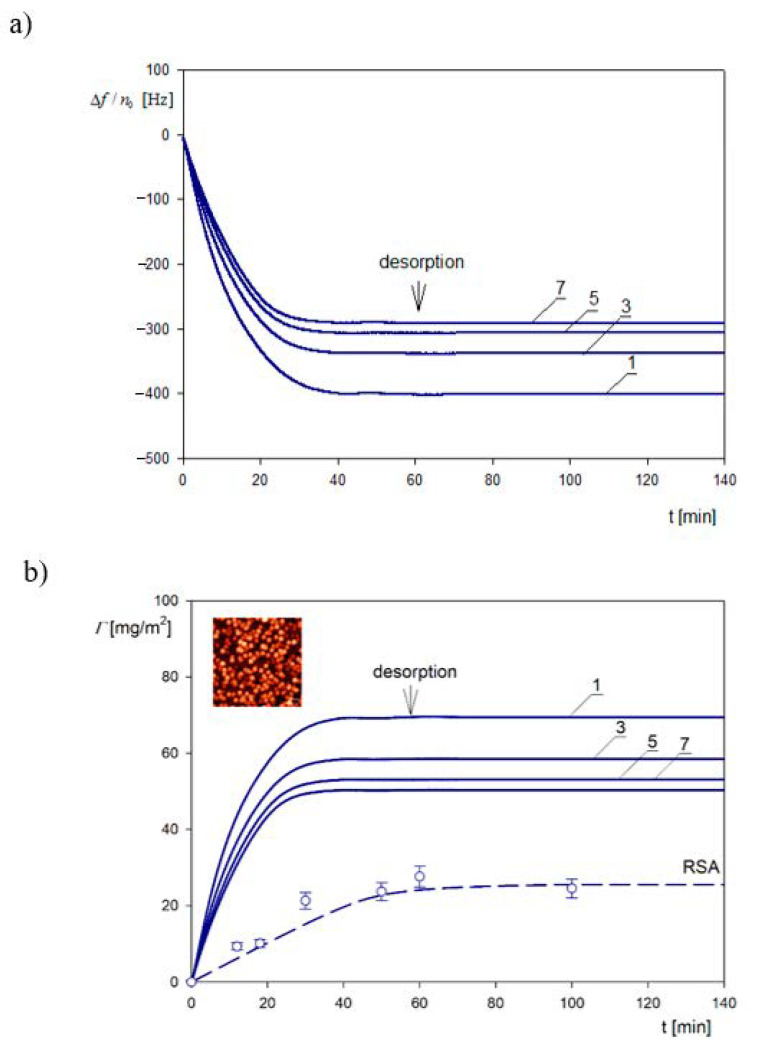
(**a**) The dependence of the normalized frequency shift $\Delta f/n_{0}$ (for the overtones 1,3,5,7) on the deposition time; silica sensor; (**b**) the kinetics of particle deposition expressed as the dependence of the coverage on the deposition time; A70 suspension, *I* = 0.01 M, pH 4.0, *Q* = 2.5 × 10^−3^ cm^3^ s^−1^, bulk concentration 10 mg L^−1^. The solid lines show the results calculated from Equation (67), the points represent the coverage derived from AFM and the dashed line shows the theoretical results calculated using the RSA model. AFM image of the particle monolayer at the sensor is shown in the inset. Reprinted (adapted) with permission from Ref. [50], https://pubs.acs.org/doi/10.1021/acs.analchem.0c03115. Copyright **©** (2020) American Chemical Society.

A primary QCM kinetic run performed for the A70 particles is shown in Figure 11a. One can see that $\Delta f/n_{0}$ abruptly decreases with the time for all overtones attaining stationary values at the time of ca. 40 min. Analogously, as in Figure 9, the decrease in $\Delta f/n_{0}$ depends on the overtone number and is equal to −400 Hz for the first overtone (fundamental frequency) and −291 Hz for the 7th overtone. It was also argued in Ref. [50] that there was no desorption of particles because the changes in $\Delta f/n_{0}$ were negligible upon switching to the pure electrolyte flow.

These primary frequency shifts were converted to the QCM coverage $\Gamma_{Q}$ expressed in mg m^−2^ using Equation (67). The results are shown in Figure 11b and compared with theoretical calculations derived from the general random sequential (RSA) approach. One can observe that the QCM coverage is considerably larger than the RSA coverage with the $\frac{\Gamma_{Q}}{\Gamma_{}}$ ratio attaining ca. 10 for short times and the first overtone. However, the ratio of the stationary (plateau) values of the coverage attained for longer times significantly decreases to the value of 3.5. It is also worth mentioning that the theoretical RSA results shown in Figure 11b are in agreement with the experimental data stemming from the ex situ AFM imaging of the particle monolayers on the sensor.

**Figure 12:**
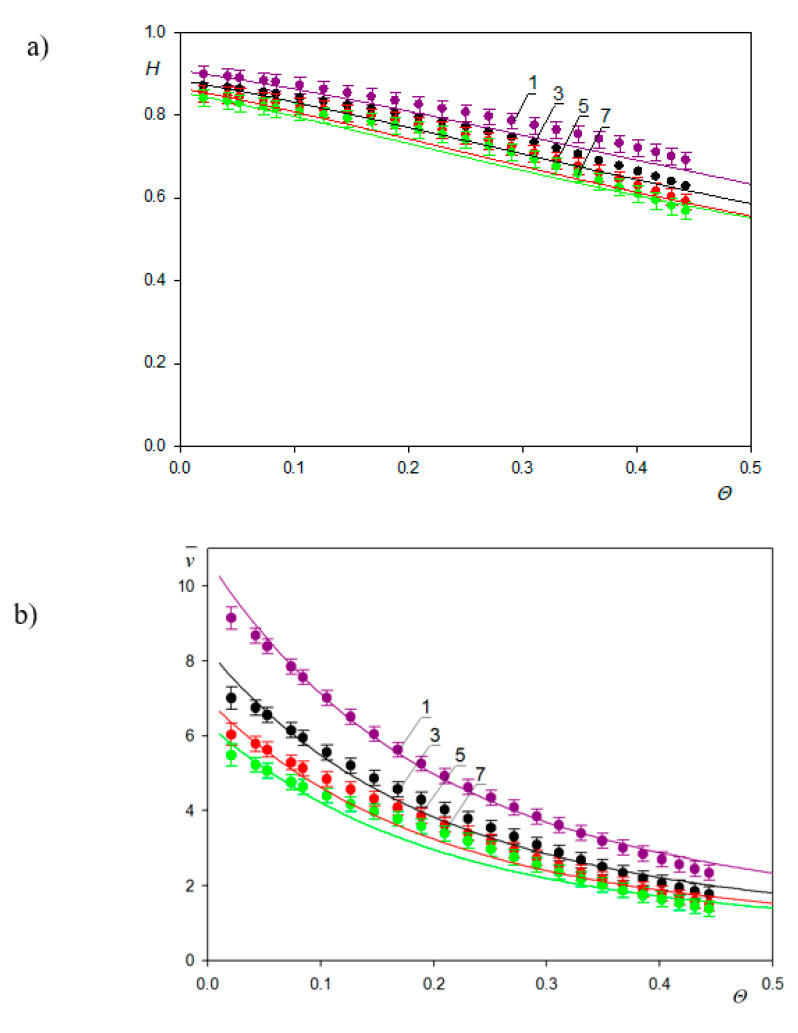
(**a**) The hydration function $H=1-\frac{\Gamma_{d}}{\Gamma_{Q}}$ for various overtones 1,3,5,7; (**b**) the function $\bar{v}$ for various overtones 1,3,5,7. The points represent the experimental data for the A70 suspension; silica sensor, pH 4.0, *I* = 0.01 M. The solid lines denote the theoretical results calculated from Equations (61), (63) and (71) and Equations (61), (63) and (72). Reprinted (adapted) with permission from Ref. [50] https://pubs.acs.org/doi/10.1021/acs.analchem.0c03115. Copyright © (2020) American Chemical Society. Further permissions related to the material excerpted should be directed to the ACS.

Using the QCM and the RSA results, the water factor and the *H* and $\bar{v}$ functions were calculated in Ref. [48]. They are plotted in Figure 12a as a function of the absolute particle coverage *Θ*. As can be seen, the *H* function in the limit of low coverage approaches 0.89 ± 0.02 and 0.84 ± 0.02, for the first and the 7th overtone, respectively. These values correlate with those of Gillissen et al. [44], who reported *H* = 0.9 for liposomes adsorbing on titania in the limits of low coverage. It is also shown in Figure 12a that for larger coverage, the hydration function monotonically decreases. These experimental data were theoretically interpreted in Ref. [50] using Equation (71), considering that $\bar{\delta }$ = 7.1 and 2.7 for the 1st and the 7th overtone. In the limit of negligible particle coverage, it is predicted that *H* = 0.95 and 0.89 for the first and the 7th overtone, respectively, which is in agrement with experimental data.

However, the calculation of the $\bar{v}$ function from Equation (72) showed that for the A70 suspension, the values should be equal to 24 and 9 in the limit of low coverage for the first and 7th overtones, respectively, considerably exceeding the experimental values. This discrepancy was interpreted in Ref. [40] as being due to the finite adhesion strength, which may allow for particle oscillatory motion. The significance of this effect is controlled by the adhesion constant *C* given by Equation (61), which depends on the work of the adhesion. Assuming a soft contact regime, theoretical results of the coverage were calculated from Equations (61) and (63) and the $\bar{v}$ function from Equation (72) shown as solid lines in Figure 12b.

A reasonable agreement with experimental data for the overtones 1–7 was attained for the value of the adhesion constant equal to $c_{a}$= 0.0082 calculated using a plausible value of the Hamaker constant and the minimum approach distance of the A70 particles to the sensor of 1 nm.

**Figure 13:**
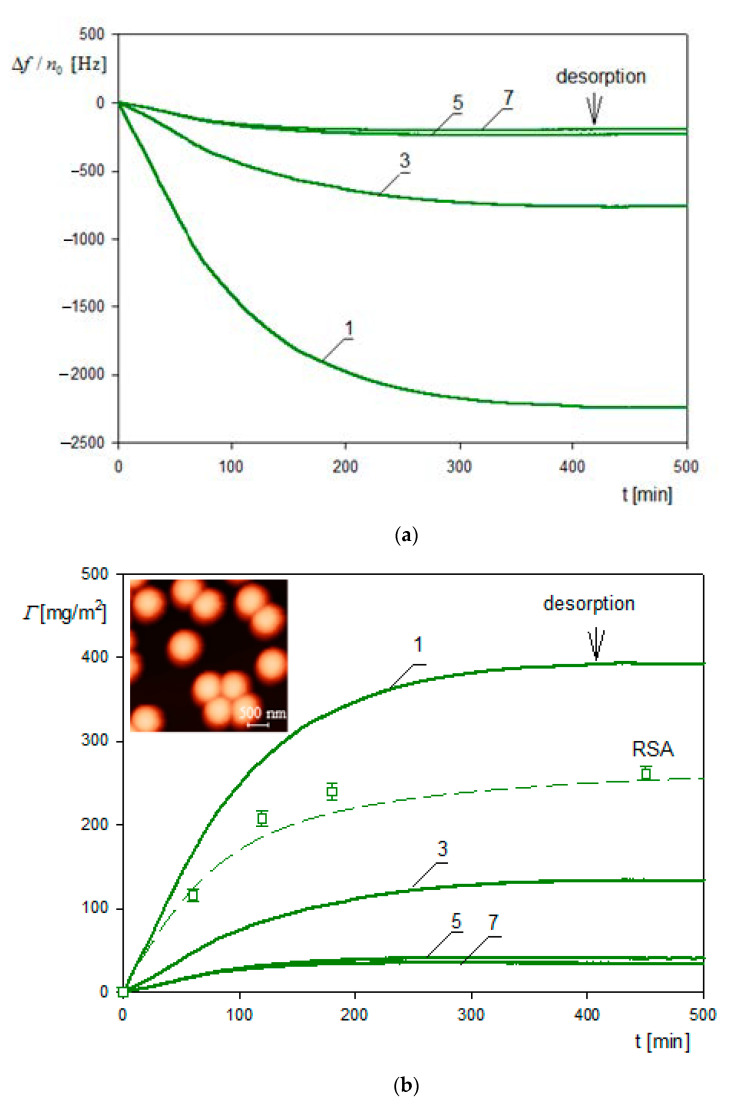
(**a**) The dependence of the normalized frequency shift $\Delta f/n_{0}$ (for the overtones 1,3,5,7) on the deposition time; silica sensor. (**b**) The kinetics of particle deposition. A800 suspension, bulk concentration 500 mg L^−1^. The AFM overage is shown by the square points, the dashed lines show the theoretical results derived from the RSA model. Reprinted (adapted) with permission from Ref. [50], https://pubs.acs.org/doi/10.1021/acs.analchem.0c03115. Copyright © (2020) American Chemical Society. Further permissions related to the material excerpted should be directed to the ACS.

The significance of the soft contact regime, enabling oscillatory motion, is more pronounced for larger particles, which is shown in Figure 13a [50]. One can observe that for the A800 particles, the frequency shift rapidly decreases (in absolute terms) with the overtone number attaining the plateau value of −2234, −760 and −235 Hz for the 1st, 3rd and the 5th overtones, respectively. For still larger overtones, it is practically negligible. In Figure 13b, the dependence of the coverage of the A800 particles for various overtones calculated from Equation (67) on the deposition time is plotted. One can observe that for the first overtone, the QCM coverage exceeds that derived from the RSA and AFM measurements. However, for the higher overtones, the QCM coverage becomes smaller than the dry (inertia) mass of the monolayer, which means that the *w* factor becomes smaller than unity and the *H* function becomes negative. This effect was interpreted in Ref. [48] as being due to the limited particle/sensor adhesion strength compared to the inertia force, which allows for oscillatory motion of the particles, albeit with no desorption. This significantly decreases the force transferred to the sensor, which may even become less that the inertia force exerted on the particle monolayer.

**Figure 14:**
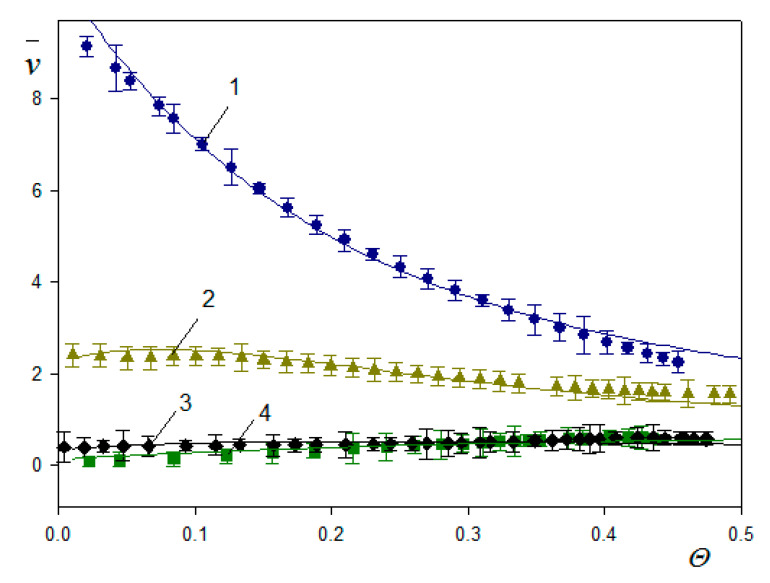
The function $\bar{v}$ for various particle suspensions: 1. A70, 2. A 140, 3. A350, 4. A800, 5. L800; the points show experimental data obtained for the 1st overtone. The theoretical results calculated from the damped oscillator model using Equations (63) and (72) are shown by solid lines. Reprinted (adapted) with permission from Ref. [50] https://pubs.acs.org/doi/10.1021/acs.analchem.0c03115. Copyright **©** (2020) American Chemical Society. Further permissions related to the material excerpted should be directed to the ACS.

The $\bar{v}$ functions derived in Ref. [50] for various particle sizes and the first overtone are shown in Figure 14. It rapidly decreases with the particle size attaining the values of 9.2 ± 0.3 to 0.4 ± 0.1 for the A70 and A350 suspension, respectively. Interestingly, for the A800 suspension, the $\bar{v}$ function in the limit of low particle coverage vanishes, which means that the $\frac{\Gamma_{Q}}{\Gamma_{}}$ ratio approaches unity. It should be mentioned that the theoretical results derived from Equations (63) and (72) assuming the rigid contact regime (solid lines in Figure 14) reasonably reflect the experimental data for various particle sizes and the entire coverage range.

Other evidence of the significant role of the adhesive contact strength between attached particles and the sensor are reported in Refs. [38,39,48]. Thus, Tarnopolsky and Freger [48] observed considerable differences in the QCM response for polystyrene and silica microparticles depending on their size and sensor chemistry (silica or gold). While polystyrene particles one micrometer in size deposited on a gold sensor produced negative frequency shifts (independent of the overtone number), the same particles on a silica sensor produced positive frequency shifts decreasing with the overtone number. Given the much smaller adhesion force for the silica sensor (due to its negative surface charge) this behavior was interpreted in terms of the soft contact model. On the other hand, for the silica particle deposition on PEI covered sensor (bearing a positive surface charge) the frequency shift was strongly negative in accordance with the freely oscillating sphere model where a rigid contact is assumed.

Analogous sensitivity of the QCM frequency shift to the sensor surface chemistry and ionic strength was observe by Olsson et al. [40] for silica particles of one micrometer size deposited on bare silica, biotinylated silica and streptavidin-coated silica. For bare silica, negative frequency shifts were only observed for overtone number below 9 and at ionic strengths exceeding 0.05 M. This was attributed to the increasing adhesion strength because of the reduction of the repulsive electrostatic interactions for larger ionic strength. For the streptavidin-coated silica, the frequency shift was strongly negative and decreased almost linearly with the overtone number analogously as reported in Ref. [46] for silica deposition on the PEI layer. These results were interpreted adopting the coupled resonance model.

In Ref. [38], a positive frequency shift for overtone numbers above 3 was determined for porous nanoncontainers produced using titania particles.

These results indicate that the adhesion forces governed to a significant extent by the sensor surface chemistry and topography (roughness) play a crucial role in the QCM measurements of both micro- and nanoparticle sized solutes. These effects become especially pronounced for larger overtones, whereas the signal acquired for the first overtone (the fundamental frequency) can yield an adequate precision of the measurements.

### 4.3. Bioparticle Deposition Kinetics

Numerous QCM-D investigations have also been performed for viruses [56,66,67,68,69,70] and proteins [54,55,56,57,58,59,60,61,62,63,64,65] using various modifications of the sensors. However, only sporadic experiments have been carried out for physiochemically well-defined systems, where the solute adsorption kinetics was also acquired using complementary methods. One such work is that of Bingen et al. [56], who performed, except for the QCM-D, in situ measurements of the solute dry mass using reflectometry. Experiments were carried out for the following solutes: the cowpea mosaic virus (CPMV), 28 nm in size, on a gold sensor, biotinylated small umilamellar vesicles (SUVs), streptavidin (SAv) and avidin (Av) on a biotinylated supported lipid bilayer (b-SLB). The solute dimensions and densities are collected in Table 1.

**Figure 15:**
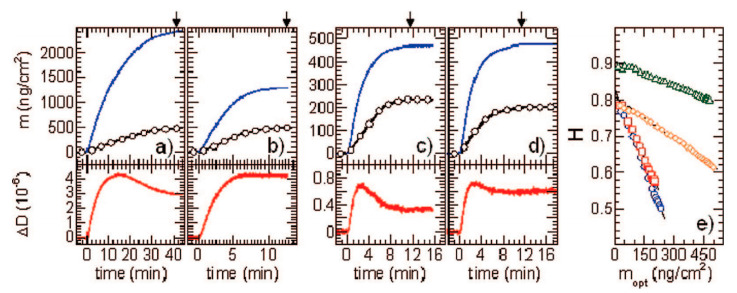
The coverage obtained from QCM (blue line) and from reflectometry (brown line with ○), the dissipation shifts (*ΔD*, red line) for various solutes: (**a**) CPMV particles on gold, (**b**) biotinylated small umilamellar vesicles (b-SUVs) on a streptavidin (SAv)-covered biotinylated supported lipid bilayer (b-SLB), (**c**) SAv on a b-SLB, and (**d**) avidin (Av) on a b-SLB. The beginning of rinsing with buffer is indicated by arrows. (**e**) The *H* hydration function for the measurements shown in (**a**–**d**): CPMV, green Δ; Sav, blue ○; Av, red □; b-SUVs, orange ◊. Reprinted (adapted) with permission from Ref. [56] Copyright © (2008) American Chemical Society.

The adsorption kinetics of these solutes derived from the reflectometric and the QCM-D measurements, which were converted to the wet coverage using Equation (67) for the 9th overtone, is shown in Figure 15. One can observe that in all cases, the QCM coverage exceeds the dry coverage derived from reflectometry by many times. Quantitatively, these results are expressed in terms of the *H* function. As seen in Figure 15e, the hydration function attains a maximum value in the limit of the low coverage of the solutes and is equal to 0.91, 0.83, 0.83, and 0.81 for CPMV, SAv, Av and SUVs, respectively (Table 1). The hydration function almost linearly decreases with the dry coverage for all solutes which qualitatively agrees with the results presented in Figure 12a for the A70 nanoparticles.

To perform a quantitative analysis, the experimental hydration function for the low coverage of these solutes are compared with the theoretical data calculated from Equation (71) with the $F_{2}(\bar{\delta })$ function pertinent to the ridged contact model calculated from Equation (73). The results collected in Table 1 show that the agreement is only satisfactory for the CPMV, where the experimental and theoretical values of *H* are 0.91 and 0.94, respectively. For proteins, the deviations are significant, suggesting that the adhesive contact was of limited strength, allowing for the oscillatory motion of the molecules. This is probable, given that the van der Walls interactions of protein molecules with substrates, because of their irregular shape and low rigidity are of limited strength. Therefore, one can expect that for protein molecules, the soft contact regime is probable, which complicates a quantitative analysis of the QCM signal because it depends on the specific surface interactions, inter alia, the electrostatic interactions.

**Figure 16:**
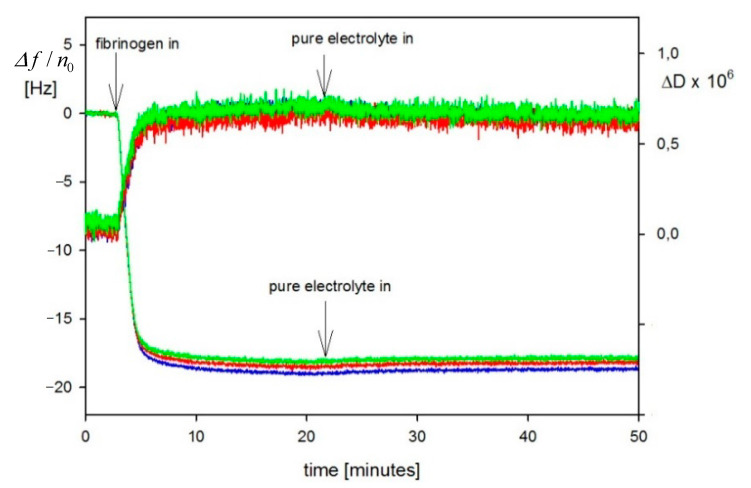
The fibrinogen adsorption/desorption run for the silica sensor, expressed as the frequency and dissipation shifts for the third, fifth and sevenths overtones (pH 3.5, ionic strength 10^−2^ M). Reprinted (adapted) with permission from Ref. [60] Copyright © Elsevier (2015).

Analogous QCM-D protein adsorption investigations focused on the determination of the hydration function under various physicochemical conditions (pH, ionic strength) have been carried out for human serum albumin [62] and fibrinogen [60,61]. An alternative method has been applied to determine the dry coverage of adsorbed proteins, which exploits the solution of the mass transfer equation derived according to the general RSA model. In Ref. [60], adsorption kinetics of fibrinogen stemming from bovine blood plasma on a bare silica sensor was studied at pH 3.5, 4.5 and 7.4 and different ionic strengths. The primary experimental run for pH 3.5 and 0.01 M NaCl is shown in Figure 16 as the dependence of $\Delta f/n_{0}$ and dissipation on time. It can be seen that both parameters were practically independent of the overtone numbers 3–7. After flushing pure electrolyte with the same flow rate, ionic strength, and pH, there was practically no change in $\Delta f/n_{0}$ and the dissipation signals, which confirms a negligible desorption of fibrinogen molecules.

**Figure 17:**
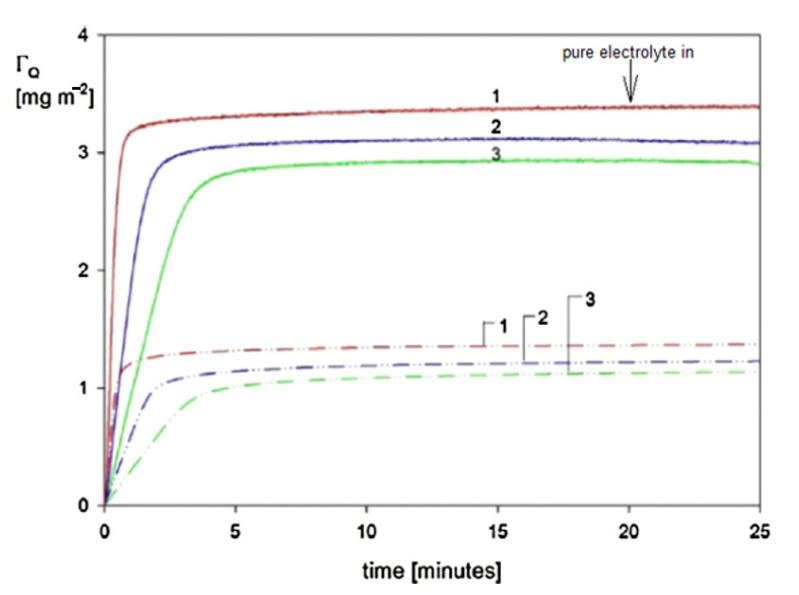
The kinetics of fibrinogen adsorption on the silica sensor, *Q* = 2.5 × 10^−3^ cm^3^ s^−1^. pH 3.5, *I* = 10^−2^ M NaCl, bulk concentration equal to: (1) 40 mg L^−1^, (2) 10 mg L^−1^, (3) 5 mg L^−1^. The dashed-dotted lines, 1–3, show the results derived from the general RSA model. Reprinted (adapted) with permission from Ref. [60] Copyright © Elsevier (2015).

Using Equation (67) the $\Delta f/n_{0}$ signal was converted to the QCM wet coverage $\Gamma_{Q}$ and is plotted in Figure 17 as a function of adsorption time. Analogously, for the A70 particles, the QCM coverage is considerably larger than the dry RSA coverage with the plateau coverage ratio exceeding two. Analogous adsorption runs were performed in Ref. [58] for other pHs and ionic strengths. 

**Figure 18:**
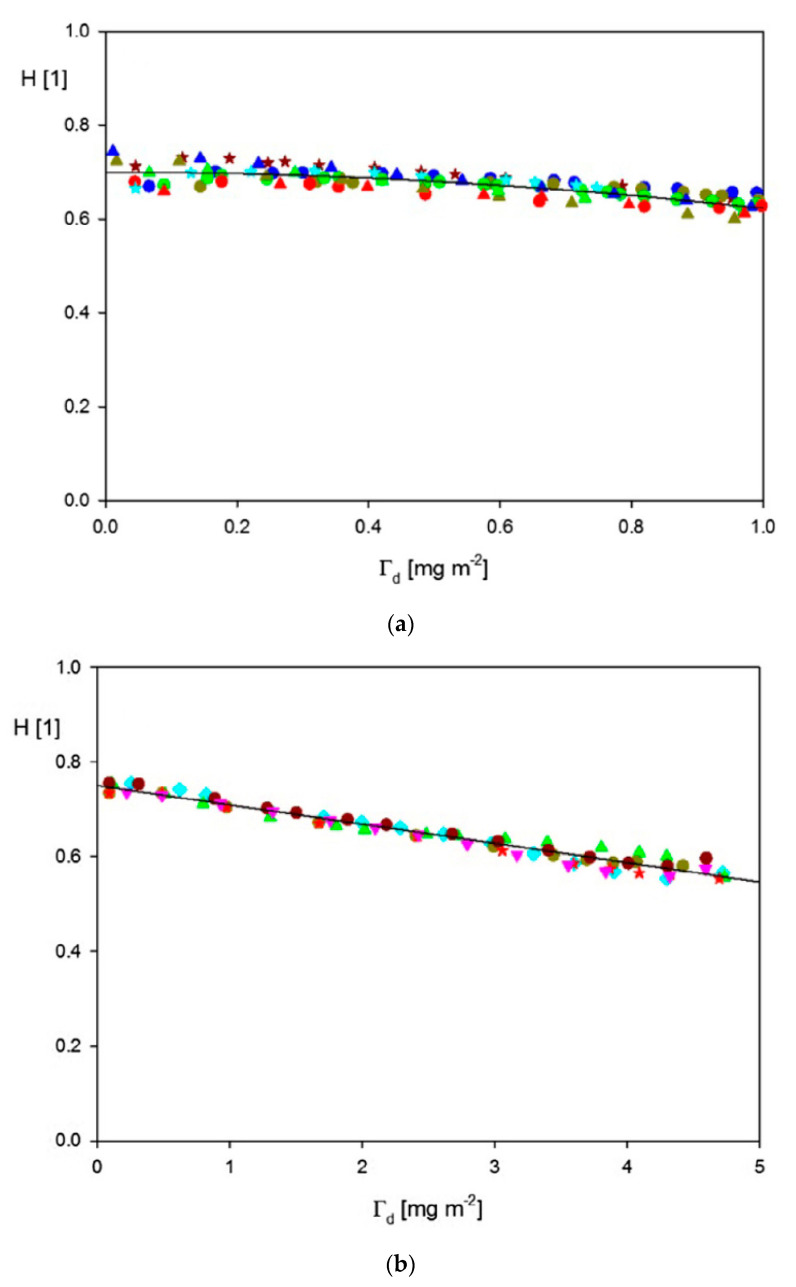
The dependence of the fibrinogen hydration function on the dry coverage calculated from Equation (71). The points denote the experimental results obtained for various bulk fibrinogen concentrations and flow rates. (**a**) pH 3.5, *I* = 10^−2^ M; (**b**) pH 7.4 (PBS), *I* = 0.15 M. The solid lines denote the polynomial interpolation of experimental results. Reprinted (adapted) with permission from Ref. [60]. Copyright © Elsevier (2015).

These kinetic runs allowed for the determination of the hydration function using Equation (71), where the dry coverage was calculated using the general RSA model. The results shown in Figure 18 confirm that universal dependencies were obtained that were independent of the bulk fibrinogen concentrations and flow rates. The low coverage values of the hydration function were equal to 0.7 and 0.75 for pH values equal to 3.5 and 7.4, respectively. 

**Figure 19:**
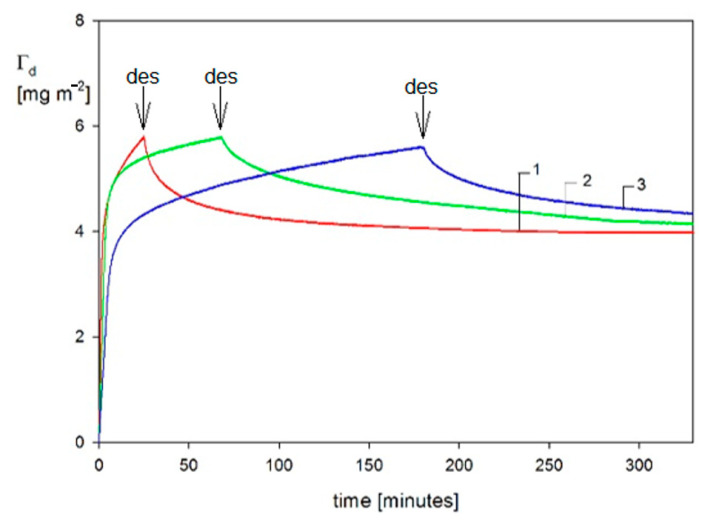
The adsorption/desorption runs expressed as the dependence of the dry coverage of fibrinogen (calculated as $\Gamma =\Gamma_{Q}\left(1-H\right)$) on the time; pH 7.4 (PBS buffer), *I* = 0.15 M, *Q* = 2.5 × 10^−3^ cm^3^s^−1^, (1) 40 mg L^−1^, (2) 20 mg L^−1^, (3) 10 mg L^−1^. The beginning of the desorption runs is indicated by arrows. Reprinted (adapted) with permission from Ref. [60]. Copyright © Elsevier (2015).

Using the hydration functions, one can convert the QCM coverage to the dry coverage, considering that $\Gamma =\Gamma_{Q}\left(1-H\right)$. However, in general, this requires the application of an inversion procedure because *H* is dependent on *Γ*. For the hydration functions shown in Figure 18, which can be fitted by a simple polynomial expression, the inversion can be analytically performed. The fibrinogen adsorption/desorption kinetics acquired from the QCM measurements using this procedure is shown in Figure 19 (pH 7.4, *I* = 0.15 M).

It is shown in Figure 19 that there was a significant fraction of reversible adsorbed fibrinogen, amounting to ca. 1.5 mg m^−2^, which could be removed after switching to the pure electrolyte flow. However, the irreversibly adsorbed fibrinogen fraction for pH 7.4 and ionic strength of 0.15 M was significantly larger and equal to 4.2 mg m^−2^. As discussed in Ref. [60], the results derived from QCM-D measurements agree with previous literature data obtained by other methods such as the TIRF and ellipsometry. It is, therefore, argued that the QCM method can furnish quantitative data pertinent to protein adsorption.

Adsorption kinetics of proteins for more complicated systems was also thoroughly investigated using the QCM-D method [63,64,65]. For example, in a recent work [61], the adsorption of fibrinogen (Fb), human serum albumin (HSA) and lysozyme (Lys) on homo PEO, PAA brushes and mixed PEO/PAA brushes was studied. The adsorption was performed on the pure and the mixed PEO/PAA brushes created in the mass ratio of 10_PEO_/90_PAA_ and 50 _PEO_/50 _PAA_ with PEO of different molar masses. This system is an example of surfaces that, due to the high grafting density resulting from the use of high concentrations of PEO and PAA in the reaction mixture for the bulk concentration of 200 mg L^−1^, was able to either inhibit protein adsorption on the homo PEO brushes or control this process in a reversible way on the mixed PEO/PAA brushes.

**Figure 20:**
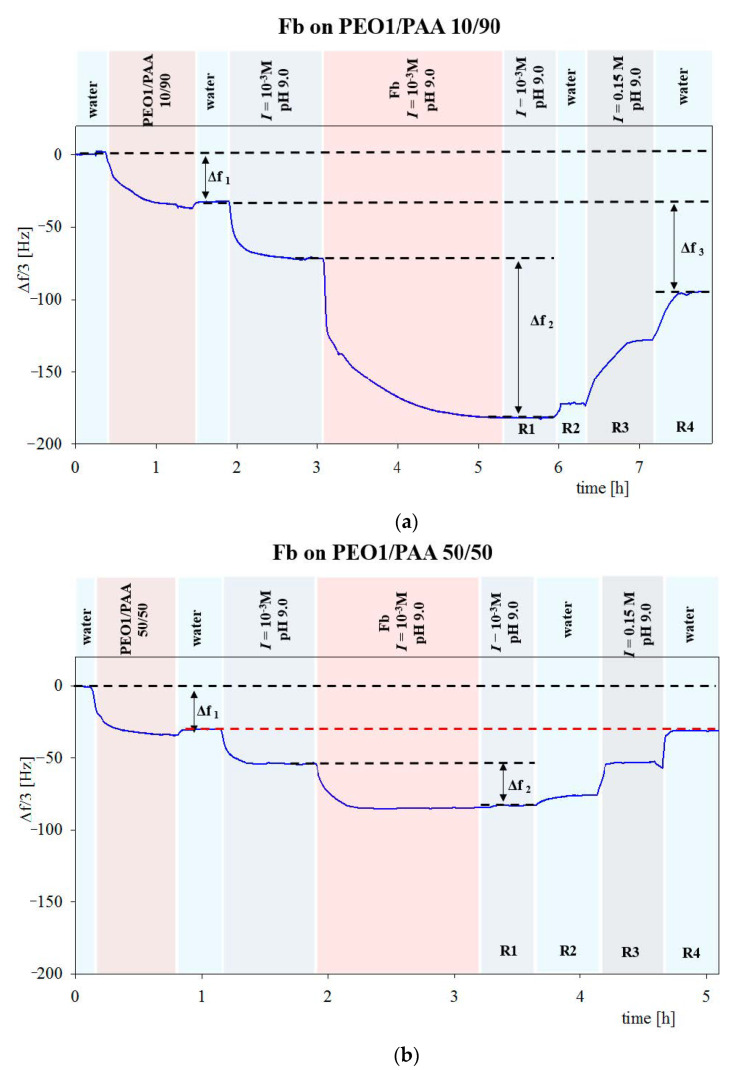
Kinetics of fibrinogen adsorption on the mixed PEO/PAA brushes monitored by QCM-D. Protein adsorption was performed at *I* = 10^−3^ M, pH 9.0, (c = 0.2 mg/mL) and desorption at *I* = 0.15M, pH 9.0. (**a**) Fb on PEO1/PAA 10/90 brushes, (**b**) Fb on PEO1/PAA 50/50 brushes. R1—Rinsing with sodium chloride solution, *I* = 10^−3^ M, pH 9.0, R2—rinsing with water, R3—desorption with sodium chloride solution *I* = 0.15 M and pH 9.0, R4- rinsing with water. Reprinted (adapted) with permission from Ref. [63] Copyright © (2018) American Chemical Society.

Fibrinogen adsorption on PEO1/PAA 10/90 polymer brushes is presented in Figure 20a. The mass of Fb adsorbed on the PEO1/PAA brush, calculated from $\Delta f_{2}$ using Sauerbrey equation was equal to 1953 ng/cm^2^. The next two shifts correspond to the rinsing with ultrapure water (R2) and the desorption step with a 0.15 M, pH 9.0 saline solution (R3). Finally, the $\Delta f_{3}$ shift corresponds to the remaining mass of Fb after the last desorption step in ultrapure water (1114 ng cm^−2^). Figure 20b shows a QCM graph of Fb adsorption on the PEO1/PAA 50/50 polymer brush. The calculated Fb mass after the adsorption step was 520 ng/cm^2^. After rinsing with water (R1−R4), a total desorption of Fb was observed.

A similar trend was observed for lysozyme, where the total desorption of the protein was observed on PEO1/PAA 50/50 brushes. However, these data were not interpreted in order to determine dry mass of adsorbed proteins from the solution of the mass transfer equation.

**Figure 21:**
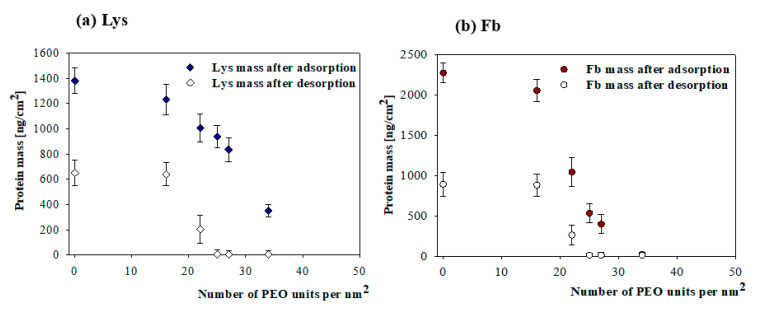
Dependence of protein mass (protein coverage) calculated from the Sauerbrey modeling of Δ*f* measured by QCM-D after adsorption step (full symbols—R1, *I* = 10^−2^ M, pH 9.0), and desorption step (open symbols—R4, *I* = 0.15 M, pH 9.0), as a function of PEO units per nm^2^ calculated from XPS measurements: (**a**) Lys, (**b**) Fb. Reprinted (adapted) with permission from Ref. [63] Copyright © (2018) American Chemical Society.

The reversibility of protein adsorption on the PEO/PAA brushes was studied as a function of PEO content and ionic strength. The mass of adsorbed proteins was slightly dependent on the ionic strength, with higher values measured at *I* = 10^−2^ M compared to 10^−3^ M. The amount of both polymers in the studied brushes was determined from XPS measurements and expressed as several PEO and PAA units per nm^2^. It was shown that the adsorbed proteins can be completely removed only from the mixed PEO/PAA brushes containing a minimum of 25 PEO units per nm^2^ (Figure 21).

## 5. Conclusions

It is shown that QCM-D response for a particle-like load acquired under a rigid contact regime can be theoretically predicted for an arbitrary particle size without using adjustable parameters. Because of the presence of the term describing the hydrodynamic force, there appears to be a non-linear dependence of the frequency and dissipation shifts on the particle dry coverage. The magnitude of this term increases with the normalized hydrodynamic boundary layer thickness $\bar{\delta }=\delta /a$, i.e., inversely proportionally to the particle size and the square root of the overtone number. As a consequence, the overtone dependence of the frequency and dissipation shifts is especially pronounced under the low coverage regime and for particles of low density.

The inversion of Equations (43) and (44) makes it possible to uniquely calculate the dry coverage of particles using the frequency or dissipation shifts acquired from QCM. However, this procedure is feasible if the $\bar{A_{i}}(\Theta )$ function describing the hydrodynamic force is explicitly known, which is currently the case for spherical particles and $\bar{\delta }$ > 2. On the other hand, for $\bar{\delta }$ < 1, one can assume with sufficient precision that $\bar{A_{i}}(\Theta )$ = 1. In this way, for $\bar{\delta }$ > 2, one obtains Equation (51), involving the dissipation shift applicable for arbitrary particle coverage, and Equation (53), involving the frequency shift, valid for the low coverage regime. These equations yield the upper limit of the QCM signal attainable for sensors providing a strong adhesion of particles.

The validity of the rigid contact regime can be uniquely assessed if the coverage derived from these formulae is compared with experimental dry coverage data acquired by supplementary experimental methods, for example, reflectometry, ellipsometry or from AFM or an electron microscope examination of the sensor.

On the other hand, theoretical analysis for the soft contact regime, where the particles attached to the sensor can execute damped oscillatory motion indicates that the QCM signal depends on the work of adhesion primarily governed the van der Waals and the electrostatic interactions. Because these interactions rapidly decrease with the minimum approach distance of the particle, it is predicted that the sensor roughness should promote a transition from the rigid to the soft contact regime. A criterion, expressed by Equation (65), is formulated, which can be used for a quantitative estimation of the transition conditions. It is predicted using this formula that the rigid contact regime can be realized for nanoparticles and for small overtone numbers provided that the adhesion strength is sufficient, i.e., for smooth and homogeneous surfaces.

These results allow thorough analysis of experimental data acquired for nanoparticles where, except for QCM, complementary SEM and AFM measurements are applied. The main results predicted by the theoretical model were confirmed, especially the abrupt decrease in the $\bar{v}$ function describing the significance of hydrodynamic forces with the particle coverage and the overtone number.

However, for particle sizes above 100 nm, the soft contact regime plays a decisive role, because the limited adhesion strengths allow for damped oscillations. As a result, the larger number overtones produce negligible QCM signals, which may yield a ratio of the QCM to the dry coverages (water factor) below unity.

Significant deviations from the rigid contact regime are also evident for bioparticles, especially protein molecules because experimentally observed frequency and dissipation shifts become little dependent on the overtone number and are much smaller than theoretically predicted from the rigid contact model. Therefore, under the present state of art, the QCM measurements in the case of bioparticles are rather specific, requiring a thorough calibration by other complementary experiments methods or theoretical modeling. However, once the system has been well-characterized, for example, the hydration function has been determined, the QCM method can be used for precise and convenient, albeit relative, measurements of bioparticle adsorption/desorption kinetic on solid substrates.

## Figures and Tables

**Table 1:** Experimental and theoretical values of *H_0_* function derived from the rigid contact model in the limit of low particle coverage.

Sample	Density [g cm^−3^]	Size [nm]	*δ/a* [1]	*H*_0_ *Experiments*	*H*_0_ *Theory*
CPMV 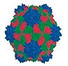	1.4	28	5.6	0.91	0.94
SAv	1.35	5.4	29	0.83	0.99
Av 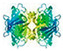	1.35	6.0	26	0.83	0.99
SUVs	1.01	30	5.3	0.81	0.95

Data from Ref. [54], CMPV—cowpea mosaic virus (biotinylated). SAv—streptavidin, Av—avidin, SUVs—umilamellar vesicles. The experimental values correspond to 9th overtone (*f_F_* = 5 × 10^6^ Hz, *δ* = 79 nm) and are calculated as *H*_0_ = 1 − *Г/Г _Q_*, the theoretical data are calculated from Equations (71) and (73) for *Г*→0.

## Data Availability

Data available in a publicly accessible repository.

## References

[1] Sau T.K., Rogach A.L., Jäckel F., Klar T.A., Feldmann J. (2010). Properties and applications of colloidal nonspherical noble metal nanoparticles. Adv. Mater..

[2] Yang S., Luo X. (2014). Mesoporous nano/micro noble metal particles: Synthesis and applications. Nanoscale.

[3] Kubiak K., Adamczyk Z., Maciejewska J., Oćwieja M. (2016). Gold nanoparticle monolayers of controlled coverage and structure. J. Phys. Chem..

[4] Oćwieja M., Adamczyk Z., Morga M., Kubiak K. (2015). Silver particle monolayers—Formation, stability, applications. Adv. Colloid Interface Sci..

[5] Maciejewska-Prończuk J. (2019). Mechanism of Formation of Noble Nanoparticle Layers at the Solid/Electrolyte Interface. Ph.D. Thesis.

[6] Miah M., Pavey K.D., Gun’ko M., Sheehan R., Cragg P.J. (2004). Observation of transient alkali metal inclusion in oxacalix[3] arenes. Supramol. Chem..

[7] Konop M., Damps T., Misicka A., Rudnicka L. (2016). Certain aspects of silver and silver nanoparticles in wound care. A Minireview. J. Nanomater..

[8] Lara H.H., Ixtepan-Turrent L., Jose Yacaman M., Lopez-Ribot J. (2020). Inhibition of Candida auris biofilm formation on medical and environmental surfaces by silver nanoparticles. ACS Appl. Mater. Interfaces.

[9] Jeong Y., Kook Y., Leea K., Koh W. (2018). Metal enhanced fluorescence (MEF) for biosensors: General approaches and a review of recent developments. Biosens. Bioelectron..

[10] Badshah M.A., Koh N.Y., Zia A.W., Abbas N., Zahra Z., Saleem M.W. (2020). Recent developments in plasmonic nanostructures for metal enhanced fluorescence-based biosensing. Nanomaterials.

[11] Chen Q., Xu S., Liu Q., Masliyah J., Xu Z. (2016). QCM-D study of nanoparticle interactions. Adv. Colloid Interface Sci..

[12] Astruc D. (2020). Introduction: Nanoparticles in catalysis. Chem. Rev..

[13] Yousefi N., Tufenkji N. (2016). Probing the Interaction between nanoparticles and lipid membranes by quartz crystal microbalance with dissipation monitoring. Front. Chem..

[14] Zembala M., Adamczyk Z. (2000). Measurements of streaming potential for mica covered by colloid particles. Langmuir.

[15] Zembala M., Adamczyk Z., Warszyński P. (2001). Influence of adsorbed particles on streaming potential of mica. Colloids Surf. A..

[16] Nattich-Rak M., Dąbkowska M., Adamczyk Z. (2020). Microparticle deposition on human serum albumin layers: Unraveling anomalous adsorption mechanism. Colloids Interfaces.

[17] Adamczyk Z., Bratek A., Szelag E., Bastrzyk A., Michna A., Barbasz J. (2009). Colloid particle deposition on heterogeneous surfaces produces by electrolyte adsorption. Colloids Surf. A: Physicochem. Eng. Asp..

[18] Adamczyk Z., Bratek-Skicki A., Żeliszewska P., Wasilewska M. (2014). Mechanisms of fibrinogen adsorption at solid substrates. Curr. Top. Med. Chem..

[19] Jin C., Glawdel T., Ren C.L., Emelko M.B. (2015). Non-linear, non-monotonic effect of nano-scale roughness on particle deposition in absence of an energy barrier: Experiments and modeling. Sci. Rep..

[20] Bratek-Skicki A., Zeliszewska P., Adamczyk Z. (2014). Human fibrinogen adsorption on latex particles at pH 7.4 studied by electrophoretic mobility and AFM measurements. Curr. Top. Med. Chem..

[21] Bratek-Skicki A., Żeliszewska P., Ruso J.M. (2016). Fibrinogen: A journey into biotechnology. Soft Matter.

[22] Cross W.M., Ma S., Winter R.M., Kellar J.J. (1999). FT-IR and SEM study of colloidal particle deposition. Colloids Surf. A Physicochem. Eng. Asp..

[23] Kollmer M., Meinhardt K., Haupt C., Liberta F., Wulff M., Linder J., Handl L., Heinrich L., Loos C., Schmidt M. (2016). Electron tomography reveals the fibril structure and lipid interactions in amyloid deposits. Proc. Natl. Acad. Sci. USA.

[24] Kleimann J., Lecoultre G., Papastavrou G., Jeanneret S., Galletto P., Koper G.J., Borkovec M. (2006). Deposition of nanosized latex particles onto silica and cellulose surfaces studied by optical reflectometry. J. Colloid Interface Sci..

[25] Toccafondi C., Prato M., Maidecchi G., Penco A., Bisio F., Cavalleri O., Canepa M. (2011). Optical properties of Yeast Cytochrome c monolayer on gold: An in situ spectroscopic ellipsometry investigation. J. Colloid Interface Sci..

[26] Höök F., Vörös J., Rodahl M., Kurrat R., Böni P., Ramsden J.J., Textor M., Spencer N.D., Tengvall P., Gold J. (2002). A comparative study of protein adsorption on titanium oxide surfaces using in situ ellipsometry, optical waveguide lightmode spectroscopy, and quartz crystal microbalance/dissipation. Colloids Surf. B..

[27] Sander M., Madliger M., Schwarzenbach R.P. (2010). Adsorption of transgenic insecticidal Cry1Ab protein to SiO2. 1. Forces driving adsorption. Environ. Sci. Technol..

[28] Wasilewska M., Adamczyk Z., Sadowska M., Boulmedais F., Cieśla M. (2019). Mechanisms of fibrinogen adsorption on silica sensors at various pHs: Experiments and theoretical modeling. Langmuir.

[29] Michna A., Pomorska A., Nattich-Rak M., Wasilewska M., Adamczyk Z. (2020). Hydrodynamic solvation of poly(amido amine) dendrimer monolayers on silica. J. Phys. Chem. C..

[30] Reimhult E., Larsson C., Kasemo B., Höök F. (2004). Simultaneous surface plasmon resonance and quartz crystal microbalance with dissipation monitoring measurements of biomolecular adsorption events involving structural transformations and variations in coupled water. Anal. Chem..

[31] Tokarczyk K., Jachimska B. (2017). Quantitative interpretation of PAMAM dendrimers adsorption on silica surface. J. Colloids Interface Sci..

[32] Bratek-Skicki A., Zeliszewska P., Adamczyk Ż. (2013). Tuning conformations of fibrinogen monolayers on latex particles by pH of adsorption. Colloids Surf. B Biointerfaces.

[33] Adamczyk Z., Sadlej K., Wajnryb E., Nattich M., Ekiel-Jeżewska M.L., Bławzdziewicz J. (2010). Streaming potential measurements of colloid, polyelectrolyte and protein deposition. Adv. Colloid Interface Sci..

[34] Adamczyk Z., Zaucha M., Zembala M. (2010). Zeta potential of mica covered by colloid particles: A streaming potential study. Langmuir.

[35] Wasilewska M., Adamczyk Z. (2011). Fibrinogen adsorption on mica studied by AFM and in situ streaming potential measurements. Langmuir.

[36] Tellechea E., Johannsmann D., Steinmetz N.F., Richter R.P., Reviakine I. (2009). Model-independent analysis of QCM data on colloidal particle adsorption. Langmuir.

[37] Johannsmann D., Reviakine I., Richter R.P. (2009). Dissipation in films of adsorbed nanospheres studied by quartz crystal microbalance (QCM). Anal. Chem..

[38] Pomorska A., Shchukin D., Hammond R., Cooper M.A., Grundmeier G., Johannsmann D. (2010). Positive frequency shifts observed upon adsorbing micron-sized solid objects to a quartz crystal microbalance from the liquid phase. Anal. Chem..

[39] Olsson A.L.J., van der Mei H.C., Johansmann D., Busscher H.J., Sharma P.K. (2012). Probing colloid-substratum contact stiffness by acoustic sensing in a liquid phase. Anal. Chem..

[40] Olsson A.L., Quevedo I.R., He D., Basnet M., Tufenkji N. (2013). Using the quartz crystal microbalance with dissipation monitoring to evaluate the size of nanoparticles deposited on surfaces. ACS Nano.

[41] Grunewald C., Schmudde M., Noufele C.N., Graf C., Risse T. (2015). Ordered structures of functionalized silica nanoparticles on gold surfaces: Correlation of quartz crystal microbalance with structural characterization. Anal. Chem..

[42] Kubiak K., Adamczyk Z., Oćwieja M. (2015). Kinetics of silver nanoparticle deposition at PAH monolayers: Reference QCM results. Langmuir.

[43] Schmudde M., Grunewald C., Risse T., Graf C. (2020). Controlling the interparticular distances of extended non-close-packed colloidal monolayers. Langmuir.

[44] Gillissen J.J.J., Jackman J.A., Tabaei S.R., Yoon B.K., Cho N.J. (2017). Quartz crystal microbalance model for quantitatively probing the deformation of adsorbed particles at low surface coverage. Anal. Chem..

[45] Gillissen J.J.J., Jackman J.A., Tabaei S.R., Cho N.-J. (2018). Numerical study on the effect of particle surface coverage on the quartz crystal microbalance response. Anal. Chem..

[46] Kananizadeh N., Rice C., Lee J., Rodenhausen K.B., Sekora D., Schubert M., Schubert E., Bartelt-Hunt S., Li Y. (2017). Combined quartz crystal microbalance with dissipation (QCM-D) and generalized ellipsometry (GE) to characterize the deposition of titanium dioxide nanoparticles on model rough surfaces. J. Hazard. Mater..

[47] Bartelt-Hunt S., Schubert E., Zhang J., Li Y. (2019). Deposition of titanium dioxide nanoparticles onto engineered rough surfaces with controlled heights and properties. Colloids Surf. B..

[48] Tarnapolsky A., Freger V. (2018). Modeling QCM-D response to deposition and attachment of microparticles and living cells. Anal. Chem..

[49] Adamczyk Z., Sadowska M. (2020). Hydrodynamic Solvent coupling effects in quartz crystal microbalance measurements of nanoparticle deposition kinetics. Anal. Chem..

[50] Adamczyk Z., Sadowska M., Żeliszewska P. (2020). Applicability of QCM-D for quantitative measurements of nano- and microparticle deposition kinetics: Theoretical modeling and experiments. Anal. Chem..

[51] Khopade A.J., Caruso F. (2002). Electrostatically assembled polyelectrolyte/dendrimer multilayer films as ultrathin nanoreservoirs. Nano Lett..

[52] Esumi K., Ichikawa M., Yoshimura T. (2004). Adsorption characteristics of poly(amidoamine) and poly(propylene imine) dendrimers on gold. Colloids Surf. A..

[53] Porus M., Clerc F., Maroni P., Borkovec M. (2012). Ion-specific responsiveness of polyamidoamine (PAMAM) dendrimers adsorbed on silica substrates. Macromolecules.

[54] Rechendorff K., Hovgaard M.B., Foss M., Zhdanov V.P., Besenbacher F. (2006). Enhancement of protein adsorption induced by surface roughness. Langmuir.

[55] Johannsmann D., Reviakine I., Rojas E., Gallego M. (2008). Effect of sample heterogeneity on the interpretation of QCM(-D) data: Comparison of combined quartz crystal microbalance/atomic force microscopy measurements with finite element method modeling. Anal. Chem..

[56] Bingen P., Wang G., Steinmetz N.F., Rodahl M., Richter R.P. (2008). Solvation effects in the quartz crystal microbalance with dissipation monitoring response to biomolecular adsorption. A phenomenological approach. Anal. Chem..

[57] Carton I., Brisson A.R., Richter R.P. (2010). Label-free detection of clustering of membrane-bound proteins. Anal. Chem..

[58] Wolny P.M., Spatz J.P., Richter R.P. (2010). On the adsorption behavior of biotin-binding proteins on gold and silica. Langmuir.

[59] Reviakine I., Johannsmann D., Richter R.P. (2011). Hearing what you cannot see and visualizing what you hear: Interpreting quartz crystal microbalance data from solvated interfaces. Anal. Chem..

[60] Kubiak K., Adamczyk Z., Wasilewska M. (2015). Mechanisms of fibrinogen adsorption at the silica substrate determined by QCM-D measurements. J. Colloid Interface Sci..

[61] Kubiak K., Adamczyk Z., Cieśla M. (2016). Fibrinogen adsorption mechanism at the gold substrate determined by QCM-D measurementsand RSA modelling. Colloids Surf. B..

[62] Adamczyk Z., Pomorska A., Nattich-Rak M., Wytrwal-Sarna M., Bernasik A. (2018). Protein adsorption mechanisms at rough surfaces: Serum albumin at a gold substrate. J. Colloid Interface Sci..

[63] Bratek-Skicki A., Eloy P., Morga M., Dupont-Gillain C. (2018). Reversible Protein adsorption on mixed PEO/PAA Polymer brushes: Role of Ionic strength and PEO content. Langmuir.

[64] Bratek-Skicki A., Cristaudo V., Savocco J., Nootens S., Morsomme P., Delcorte A., Dupont-Gillain C. (2019). Mixed polymer brushes for the selective capture and release of proteins. Biomacromolecules.

[65] Bratek-Skicki A. (2020). Design of Ultra-Thin PEO/PDMAEMA polymer coatings for tunable protein adsorption. Polymers.

[66] Eun A.J., Huang L., Chew F.T., Li S.F., Wong S.M. (2002). Detection of two orchid viruses using quartz crystal microbalance (QCM) immunosensors. J. Virol. Methods.

[67] Peduru Hewa T.M., Tannock G.A., Mainwaring D.E., Harrison S., Fecondo J.V. (2009). The detection of influenza A and B viruses in clinical specimens using a quartz crystal microbalance. J. Virol. Methods.

[68] Chen Y.S., Hung Y.C., Chiou J.C., Wang H.L., Huang H.S., Huang L.C., Huang G.S. (2010). Ultrasensitive detection of cymbidium mosaic potexvirus using a single-wall carbon nanotube-functionalized quartz crystal microbalance. Jpn. J. Appl. Phys..

[69] Gutierrez L., Mylon S.E., Nash B., Nguyen T.H. (2010). Deposition and aggregation kinetics of rotavirus in divalent cation solutions. Environ. Sci. Technol..

[70] Rayaprolu V., Manning B.M., Douglas T.R., Bothner B. (2010). Virus particles as active nanomaterials that can rapidly change their viscoelastic properties in response to dilute solutions. Soft Matter.

[71] Wan Y., Zhang D., Hou B. (2010). Determination of sulphate-reducing bacteria based on vancomycin-functionalized magnetic nanoparticles using a modification-free quartz crystal microbalance. Biosens. Bioelectron..

[72] Zhang F., Li H., Wang X., Low H.Y., Li X. (2010). Hierarchically imprinted polymer substrates for enhanced attachment of Escherichia coli. J. Colloids Interface Sci..

[73] Gabi M., Hefermehl L., Lukic D., Zahn R., Vörös J., Eberli D. (2011). Electrical microcurrent to prevent conditioning film and bacterial adhesion to urological stents. Urol. Res..

[74] Olsson A.L., van der Mei H.C., Busscher H.J., Sharma P.K. (2010). Novel analysis of bacterium-substratum bond maturation measured using a quartz crystal microbalance. Langmuir.

[75] Gutman J., Walker S.L., Freger V., Herzberg M. (2013). Bacterial attachment and viscoelasticity: Physicochemical and motility effects analyzed using quartz crystal microbalance with dissipation (QCM-D). Environ. Sci. Technol..

[76] Fulgione A., Cimafonte M., Della Ventura B., Iannaccone M., Ambrosino C., Capuano F., Proroga Y.T.R., Velotta R., Capparelli R. (2018). QCM-based immunosensor for rapid detection of Salmonella Typhimurium in food. Sci. Rep..

[77] Shan Y., Liu L., Liu Y., Harms H., Wick L.Y. (2020). Effects of electrokinetic phenomena on bacterial deposition monitored by quartz crystal microbalance with dissipation monitoring. Environ. Sci. Technol..

[78] Cama G., Jacobs T., Dimaki M.I., Svendsen W.E., Hauptmann P., Naumann M. (2010). Disposable micro-fluidic biosensor array for online parallelized cell adhesion kinetics analysis on quartz crystal resonators. Meas. Sci. Technol..

[79] Chou H.C., Yan T.R. (2010). Applying the quartz crystal microbalance technique to detect the epithelial cell tight junction integrality of caco-2 cells. Anal. Lett..

[80] Gun’ko V.M., Mikhalovska L.I., Savina I.N., Shevchenko R.V., James P.E., Tomlins S.L., Mikhalovsky S.V. (2010). Characterization and performance of hydrogel tissue scaffolds. Soft Matter.

[81] Huang X., Zauscher S., Klitzman B., Truskey G.A., Reichert W.M., Kenan D.J., Grinstaff M.W. (2010). Peptide interfacial biomaterials improve endothelial cell adhesion and spreading on synthetic polyglycolic acid materials. Ann. Biomed. Eng..

[82] Xi J., Chen J.Y., Garcia M.P., Penn L.S. (2013). Quartz crystal microbalance in cell biology studies. Biochip Tissue Chip.

[83] Johannsmann D. (2008). Viscoelastic, mechanical, and dielectric measurements on complex samples with the quartz crystal microbalance. Phys. Chem. Chem. Phys..

[84] Rechendorff K., Hovgaard M.B., Foss M., Besenbacher F. (2007). Influence of surface roughness on quartz crystal microbalance measurements in liquids. J. Appl. Phys..

[85] Levich V.G. (1962). Physicochemical Hydrodynamics.

[86] Adamczyk Z., Warszyński P. (1996). Role of electrostatic interactions in particle adsorption. Adv. Colloid Interface Sci..

[87] Elimelech M., Gergory J., Jia X., Williams K.A. (1995). Particle Deposition and Aggregation.

[88] Adamczyk Z. (2003). Particle adsorption and deposition: Role of electrostatic interactions. Colloid Interface Sci..

[89] Adamczyk Z., Hubbard A. (2017). Particles at Interfaces: Interactions, Deposition, Structure.

[90] Evans J.W. (1993). Random and cooperative sequential adsorption. Rev. Mol. Phys..

[91] Adamczyk Z., Weroński P. (1996). Random sequential adsorption of spheroidal particles: Kinetics and jamming limit. J. Chem. Phys..

[92] Adamczyk Z. (2000). Kinetics of diffusion-controlled adsorption of colloid particles and proteins. J. Colloid Interface Sci..

[93] Talbot J., Tarjus G., Van Tassel P.R., Viot P. (2000). From car parking to protein adsorption: An overview of sequential adsorption processes. Colloids Surf. A.

[94] Weroński P. (2005). Application of the extended RSA models in studies of particle deposition at partially covered surfaces. Adv. Colloid Interface Sci..

[95] Von Smoluchowski M. (1916). Drei vorträge fiber diffusion, Brownsche bewegung und koagulation von kolloidteilchen. Z. Phys..

[96] Bowen B.D., Levine S., Epstein N. (1976). Fine particle deposition in laminar flow through parallel-plate and cylindrical channels. J. Colloid Interface Sci..

[97] Zhang M., Soto-Rodriguez J., Chen I.-C., Akbulut M. (2013). Adsorption and removal dynamics of polymeric micellar nanocarriers loaded with a therapeutic agent on silica surfaces. Soft Matter.

[98] Ruckenstein E. (1978). Reversible rate of adsorption or coagulation of brownian particles—Effect of the shape of the interaction potential. J. Colloid Interface Sci..

[99] Ricci S.M., Talbot J., Tarjus G., Viot P. (1992). Random sequential adsorption of anisotropic particles. II. Low coverage kinetics. J. Chem. Phys..

[100] Hinrichsen E.L., Feder J., Jossang T. (1986). Geometry of random sequential adsorption. J. Stat. Phys..

[101] Sauerbrey G. (1959). Vervendung von Schwingquartzen zor Waegung duenner Schichten und zur Mikrowaegung. Z. Phys..

[102] Ekiel-Jeżewska M.J., Adamczyk Z., Bławzdziewicz J. (2019). Streaming current and effective ζ-potential for particle-covered surfaces with random particle distributions. J. Phys. Chem. C.

